# Validity and reliability of the Thai version Consumer Assessment of Healthcare Providers and Systems In-Centre Hemodialysis (Thai CAHPS-ICH) survey for maintenance hemodialysis patients

**DOI:** 10.1186/s12882-026-05042-5

**Published:** 2026-06-04

**Authors:** Jirawat Phuphanitcharoenkun, Phoom Narongkiatikhun, Vuddhidej Ophascharoensuk

**Affiliations:** Division of Nephrology, Department of Internal Medicine, Faculty of Medicine, Chiang Mai University, Chiang Mai, Thailand

**Keywords:** Patient-reported experience measure, Maintenance hemodialysis, Thai CAHPS-ICH survey

## Abstract

**Background:**

Patient-reported experience measures (PREMs) are important tools for evaluating the quality of care from patients’ perspectives. Nevertheless, no validated PREMs exist for maintenance hemodialysis (MHD) patients in Thailand. This study aimed to develop a Thai version of the Consumer Assessment of Healthcare Providers and System In-center Hemodialysis (CAHPS-ICH) survey and assess its psychometric properties, specifically its validity and reliability.

**Method:**

We conducted the cross-sectional study, using Total Population Sampling among adult patients (≥ 18 years) receiving MHD at the Chiang Mai University Hospital hemodialysis center. The original English version CAHPS-ICH^®^ survey was translated into Thai using the forward-backward translation method and cognitive debriefing. Confirmatory Factor Analysis (CFA) was employed to evaluate construct validity. Correlation analysis (using Pearson’s and Spearman’s coefficients) was used to assess the relationship between each domain (Nephrologists’ communication and caring [NCC], quality of dialysis center cares and operation [QoC], and providing information to patients [PI]) and the global rating scales. Internal consistency and test-retest reliability were assessed using Cronbach’s alpha coefficient (α) and intraclass correlation coefficient (ICC), respectively.

**Results:**

A total of 189 patients were recruited. The NCC domain CFA model showed a mixed fit (RMSEA = 0.137; CFI = 0.945; SRMR = 0.041). In contrast, the QoC and PI domain CFA models demonstrated an acceptable fit (RMSEA = 0.098; CFI = 0.844; SRMR = 0.071 and RMSEA = 0.066; CFI = 0.843; SRMR = 0.070, respectively). The NCC domain exhibited the strongest correlation with the doctor global rating scale (*r* = 0.60) and displayed good internal consistency (α = 0.86). The QoC domain indicated positive correlations with the staff and center rating scales (*r* = 0.65 and 0.58, respectively) and demonstrated acceptable internal consistency (α = 0.78). The PI domain showed a moderate association with the center global rating scales (*r* = 0.31), but its internal consistency was not demonstrated (α = 0.53). Most multi-item scale questions showed satisfactory test-retest reliability (ICC = 0.50–0.85).

**Conclusion:**

Despite the mixed fit observed in the NCC domain CFA, the overall “Thai CAHPS-ICH” survey demonstrates acceptable reliability and concurrent validity among MHD patients at Chiang Mai University Hospital. This validation is a crucial first step toward establishing a patient-centered quality metric for hemodialysis centers in Thailand.

**Trial registration:**

This study was reviewed and approved by the Ethics Committee of Chiang Mai University (Ethical approval number: MED-2565-09133).

**Supplementary Information:**

The online version contains supplementary material available at 10.1186/s12882-026-05042-5.

## Background

**Fig. 1:**
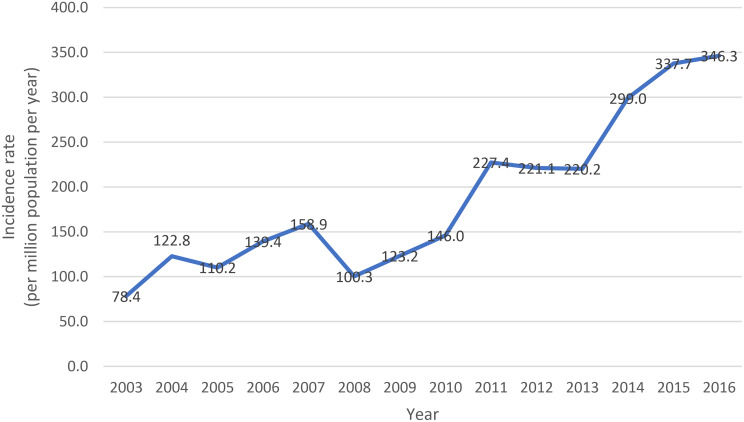
Trends in the incidence rate of ESKD in Thailand, 2003–2016. (Adapted from United States Renal Data System: 2018 USRDS annual data report. Volume II: International comparisons. Available at: https://www.niddk.nih.gov/-/media/Files/USRDS/2018-Previous-ADR/Reference-Tables/esrd_ref_n_international_2018.xlsx. Accessed November 22, 2025.) [2]

In Thailand, there has been a growing demand for renal replacement therapy (RRT) among patients with end-stage kidney disease (ESKD). In 2016, the incidence of ESKD in Thailand was 346 patients per million people per year, compared to 100 per million people per year in 2008 (Fig. 1) [1, 2]. Consequently, it is imperative to assess patient outcomes post-hemodialysis to enhance the quality of care. Various aspects of the “quality pyramid” have been discussed in terms of quality in ESKD programs, including the higher hierarchies of care such as mortality, hospitalization, patient experience and health-related quality of life (HRQoL) [3].

The patient-reported experience measures (PREMs) and patient-reported outcome measures (PROMs) have been established to evaluate the quality of care from patients’ perspectives and their HRQoL. These measures aim to improve the quality of care and clinical effectiveness in healthcare delivery. Several PROMs and PREMs have been developed and validated for adults with kidney diseases, including the Consumer Assessment of Healthcare Providers and Systems In-Center Hemodialysis (CAHPS-ICH) survey and the Consumer Quality Index of Chronic Dialysis, which are validated renal-specific PREMs [4–6].

The CAHPS-ICH survey is a particularly significant instrument because it is the first patient-reported quality metric added to the U.S. End-Stage Renal Disease Quality Incentive Program (ESRD QIP). The US Centers for Medicare & Medicaid Services (CMS) initiated national implementation of this survey in 2014 [7]. Due to its status as a mandatory quality metric, the CAHPS-ICH survey has the most extensive body of research data available [6, 8–10] and has been translated into numerous languages, including Spanish, Chinese, and Samoan [11]. The CAHPS-ICH survey comprises 58 items that assess three domains: Nephrologist Communications and Caring (NCC; 6 items), Quality of Care and Operation (QoC; 17 items), and Patient Involvement (PI; 9 items), along with three global rating scales measuring the nephrologists, dialysis center staff, and the overall dialysis center [6]. The original questionnaire is publicly available at https://ichcahps.org/Survey-and-Protocols. While many studies have shown an association between survey scores and patient and hemodialysis facility characteristics [6, 8–10], the relevance of the survey to clinical outcomes has yet to be definitively demonstrated [12].

In Thailand, no validated PREMs are currently available for maintenance hemodialysis (MHD) patients. Furthermore, despite the availability of many validated PREMs related to quality of care internationally, none of these tools have been formally adopted and implemented within the Thai public health system. Therefore, this study represents the first phase of our project, which is aimed at developing a PREM for Thai MHD patients by validating the CAHPS-ICH survey and evaluating its psychometric properties, including validity and reliability, in a Thai context. Additionally, the long-term objective of this project is to implement the validated Thai CAHPS-ICH survey as a standardized performance measurement tool for evaluating the quality of care provided by hemodialysis centers across Thailand.

## Methods

### Translational process

The cross-cultural adaptation of English version of CAHPS-ICH survey commenced following permission approval, guided by the guideline for translation and cultural adaptation [13]. First, permission request was submitted via email to David I. Lewin, the Health Communications Specialist/Manager of Copyrights & Permissions, Agency of Healthcare Research and Quality (AHRQ), who is responsible for the international requests for the CAHPS survey usage. Second, two independent forward translations of the English version of the CAHPS-ICH to Thai were conducted. The first translator was a specialist physician (internal medicine) currently practicing at our academic hospital, and the second was a university instructor from the Faculty of Humanities who lacked a clinical background, ensuring translation diversity. To reconcile any discrepancies between these two translations, they were discussed and resolved by an expert committee. Third, a backward translation into English was performed by a language expert, who was blinded to the original English version. Fourth, all the translations and any remaining discrepancies were reviewed by the expert committee, comprising nephrologists, hemodialysis nurses, and language professionals, until a consensus was reached for all the items. Moreover, the committee compared the back-translated version with the original to identify any inconsistent conception and resulting in the preliminary version of the Thai CAHPS-ICH. Following this, the committee made necessary corrections and adjustments, resulting in the preliminary version of the “Thai CAHPS-ICH” questionnaire. To assess item understandability, clarity, and potential embarrassment, the preliminary Thai CAHPS-ICH survey was tested with 10 subjects using a cognitive debriefing method. Following this qualitative testing, the expert committee made necessary corrections and adjustments based on the feedback, producing the final version of the “Thai CAHPS-ICH” questionnaire. While the study primarily adhered to the AHRQ’s standardized validation protocol for international CAHPS adaptations, the qualitative rigor of our expert review and cognitive debriefing processes addresses the core components of content relevance and comprehensibility. We acknowledge that a formal quantitative Content Validity Index (CVI) and a comprehensive adherence to the COSMIN checklist were not part of this initial validation phase; however, these standards are prioritized for our future refinement of the instrument to align with global psychometric reporting practices.

### Thai CAHPS-ICH survey

Similar to the original CAHPS-ICH survey, this Thai version comprises 58 items grouped into three composite domains— NCC, QoC and PI domain—and includes three global rating scales (rating of nephrologist, rating of hemodialysis center staff, and rating of hemodialysis center). The majority of items utilize a standardized 4-point Likert scale (e.g., Never, Sometimes, Usually, or Always) to measure the frequency of patient experiences. In contrast, the three global rating scales are measured on an 11-point scale ranging from 0 (Worst possible) to 10 (Best possible). For standardization and interpretation, the raw scores for the three composite domains are linearly transformed into a 0 to 100-point scale. A higher score indicates a more favorable patient experience, with the final domain score calculated as the average of the transformed item scores within that specific domain.

### Study designs and population

We conducted a cross-sectional study at the hemodialysis center of Chiang Mai University Hospital. This study enrolled participants who met the following inclusion criteria: being 18 years of age or older, of Thai nationality, and having received maintenance hemodialysis for at least 3 months at Chiang Mai University Hospital. The exclusion criteria were patients who were unable to communicate, who had cognitive impairment, who required assistance (defined as patients with a Karnofsky Performance Status Scale score below 50), who received temporary hemodialysis, or who received regular hemodialysis from another hemodialysis center. The sampling method used was Total Population Sampling. All eligible patients who met the specified inclusion criteria were invited to participate in the study.

### Data collection

Ethical approval for the study was granted by the Ethics Committee of Chiang Mai University (MED-2565-09133). Eligible patients were identified and approached by research staff. Once written informed consent was obtained, participants were administered the ‘Thai CAHPS-ICH’ survey. The staff’s role was strictly limited to distributing and collecting the questionnaires and they were explicitly instructed not to intervene in the survey response process. For independent individuals, questionnaires had to be self-completed and returned during the next hemodialysis session. Due to the length of the questionnaire, a specific time limit for completion was not set to minimize respondent stress and ensure candid responses. Participants were allowed to complete the survey at home. Minor assistance, defined as aid from caregivers or next-of-kin in reading questions and recording answers for patients unable to read or write, was permitted. Questionnaires completed by someone else or with a response rate below 50% were excluded from the analysis. The questionnaire was re-administered four weeks later using the same methods to assess test-retest reliability.

Patient demographic data were collected, and hemodialysis and laboratory parameters were recorded. The weekly dosage of all erythropoietin stimulating agents (ESAs) was converted to erythropoietin alfa dosage using a conversion Table [14]. Trained nurses collected all data via case record forms.

### Statistical analysis and psychometric evaluation

We determined the survey response rate and used means, medians, standard deviations, frequency tables, and rates to describe the patient characteristics. Ceiling and floor effects were utilized to assess all Likert scales in the survey, considering effects significant if items scored by responders exceeded 15% for each item. The psychometric properties of the survey were evaluated by assessing construct validity and reliability (internal consistency and test-retest reliability). To evaluate construct validity, we compared the three domains in the “Thai CAHPS-ICH” survey (NCC, QoC, PI) with global rating scales for nephrologists, hemodialysis staff, and the hemodialysis center. The normality of the data was assessed using the Shapiro-Wilk test. For variables that followed a normal distribution, Pearson’s correlation coefficient was used. For variables with a non-normal distribution, Spearman’s rank correlation was applied to evaluate the associations between survey items and global ratings. Correlation coefficients between 0.1 and 0.29, 0.3–0.49, and 0.5 or above are considered small, medium, and large associations, respectively [15]. We performed Confirmatory Factor Analysis (CFA) to test the hypothesis that the observed variables loaded onto each of the three hypothesized domains (constructs). Three separate CFA models were executed, with model fit assessed using three key statistics: the Root Mean Square Error of Approximation (RMSEA), the Comparative Fit Index (CFI), and the Standardized Root Mean Square Residual (SRMR). Model fit was interpreted using standard criteria: an RMSEA value of < 0.08, 0.08–0.1, > 0.1 indicated a good fit, mediocre fit, and poor fit, respectively; a CFI of ≥ 0.95 indicated a good fit; and an SRMR value of < 0.05 indicated a good fit, with < 0.08 considered acceptable [16]. Moreover, in each CFA model, we assessed the Factor Loading for every item to indicate how well it represents its corresponding domain. A Factor Loading value of ≥ 0.50 was used as the minimum acceptable threshold for practical significance [17]. The internal consistency reliability of each domain was measured using Cronbach’s alpha (α) coefficient, with values ≥ 0.7–0.8, > 0.8–0.9, and > 0.9 considered acceptable, good, and excellent for aggregate data, respectively. The intraclass correlation coefficient (ICC) was used to evaluate test-retest reliability. A Two-Way Mixed Effects Model for Absolute Agreement was utilized, with values < 0.5, 0.5-<0.75, 0.75-<0.9, and 0.9-1 indicating poor, fair, good, and excellent agreement, respectively [18]. This repeatability was evaluated using questionnaires at the start and fourth week after enrollment. This interval was chosen to minimize memory recall bias while ensuring the clinical stability of the participants’ experiences [19]. Statistical software used in this study was SPSS, version 17.0 (SPSS Inc., Chicago, IL, USA).

### Sample size

The sample size determination was based on both reliability analysis and factor analysis requirements. First, according to the sample size estimation for reliability studies, the minimum required sample size was 118 to achieve alpha errors of 0.05 and a statistical power of 0.80 [20]. Second, for CFA, the recommended sample size is typically based on the ratio of participants to items (N: p ratio), where a minimum ratio of 5:1 is often cited [21]. Given that 32 items from the 58-item Thai CAHPS-ICH survey were designated for inclusion in the CFA, a minimum sample size of 5 × 32 = 160 was required for factor analysis. In our hemodialysis center, the total number of MHD patients was at least 300. The anticipated response rate was approximately 40%, based on findings from a previous study [22]. Therefore, to ensure maximum statistical power and satisfy the requirements for both reliability and factor analysis (minimum participants = 160), we aimed to recruit all eligible patients to complete the questionnaire.

## Results

**Fig. 2:**
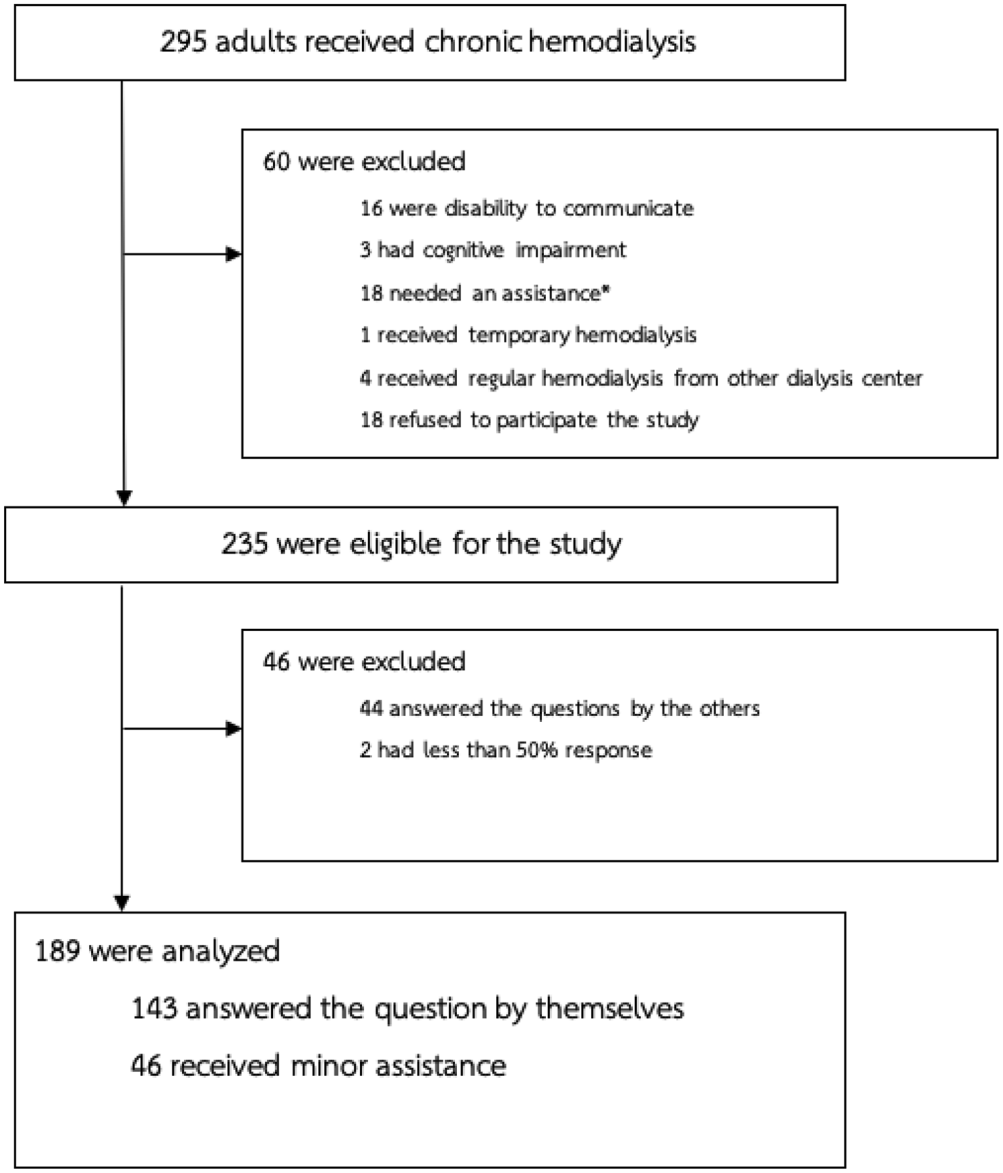
Flow diagram of the study participants. *Patient who needed assistance is defined as those with a Karnofsky Performance Scale score below 50

During September and December 2022, there were 295 MHD patients at the hemodialysis center of Chiang Mai University Hospital. As depicted in Figs. 2 and 106 patients were excluded. One hundred eighty-nine patients were included in the analysis. The survey response rate was 80.43%. Patient characteristics are shown in Table 1. The mean age was 58.28 ± 14.87 years. The majority of patients were aged between 55 and 74 years (55.56%). The median hemodialysis vintage was 39 months (IQR; 18,72). Among the 189 patients included in the analysis, 143 (75.66%) completed the questionnaire themselves. Our “Thai CAHPS-ICH” survey is provided in the supplementary appendix.

**Table 1:** Patient characteristics at baseline

Characteristics	*N* (%) (Total = 189)
**Age (years old)**	
18–34 35–54 55–74 75 or more	12 (6.35) 53 (28.04) 105 (55.56) 19 (10.05)
**Sex**	
Male Female	107 (56.61) 82 (43.39)
**BMI (kg/m** ^**2**^ **)**	
< 18.5 18.5–22.9 23–25 > 25	23 (12.17) 72 (38.10) 37 (19.58) 57 (30.16)
**Hemodialysis Vintage**	
3 months – less than 1 year 1 – less than 5 years 5 years or more	31 (16.40) 100 (52.91) 58 (30.69)
**Education**	
Elementary School Junior high school Senior high school Bachelor Master’s degree or more	56/187 (29.95) 17/187 (9.09) 30/187 (16.04) 63/187 (33.69) 21/187 (11.23)
**Survey response**	
Self-complete Reading the question Writing down the given answer Translating the question into local language	143 (75.66) 43 (22.75) 25 (13.23) 4 (2.12)

The ceiling and floor effects are shown in Table 2. All the 10-point global rating scales (Q8, Q32, and Q35) exhibited significant ceiling effects (ranging from 39.89 to 48.91%). Ceiling effects were also observed in most composites of the NCC and QoC domains, with exceptions noted for “Q24 staff manage problem” (12.07%) and “Q43 satisfied with way problems handled” (1.14%). None of the questions demonstrated significant floor effects (0–13.89%).

**Table 2:** Ceiling and floor of the Thai CAHPS-ICH

Thai CAHPS-ICH	Baseline		1 month	
	%Ceiling effect	%Floor effect	%Ceiling effect	%Floor effect
Q3 Doctor listens carefully	59.57	1.06	52.69	0.60
Q4 Doctor explains things	38.67	2.76	42.17	1.81
Q5 Doctor shows respect	47.49	0	44.85	0.61
Q6 Doctor spends enough time	42.54	0.55	48.19	0
Q7 Doctor cared about you	48.62	1.10	46.99	0.60
Q8 Nephrologists’ communication and caring	39.89	0	43.71	0
Q10 Staff listen carefully	64.84	0	59.52	0
Q11 Staff explain in a way that is easy to understand	55.25	0	54.76	0
Q12 Staff show respect	51.65	0	55.36	0
Q13 Staff spend enough time	57.07	0	52.69	0.60
Q14 Staff cared about you	57.84	0	55.95	0
Q15 Staff makes you comfortable	61.41	0.54	62.50	2.38
Q21 Staff insert needle without pain	34.07	1.65	36.42	0
Q22 Staff check you closely	57.61	0.54	52.07	1.18
Q24 Staff manage problems	12.07	0	17.18	0.61
Q25 Staff professional	64.77	0	56.36	1.82
Q27 Staff explain tests	32.42	4.95	31.52	4.24
Q32 Quality of Dialysis Center Care and Operations	46.70	0	46.43	0
Q33 On machine within 15 min	32.78	13.89	35.33	10.78
Q34 Center clean	67.21	0.55	65.87	0
Q35 Providing Information to Patients	48.91	0	50.90	0
Q43 Satisfied with way problems handled	1.14	0	1.23	1.23

Table 3 displays the results of the validity assessment, presenting the correlations between survey domains and global rating scales. The NCC domain demonstrated a strong association with the global rating scale of nephrologists (*r* = 0.60). Similarly, the QoC scale exhibited a strong association with the global rating scales of staff and center (*r* = 0.65 and *r* = 0.58, respectively). Additionally, a moderate association was observed between the PI scale and center global rating scale (*r* = 0.31).

**Table 3:** Correlations between scales and global rating scales

Scale domains	Doctor rating scale	Staff rating scale	Center rating scale
Nephrologists’ Communication and Caring (NCC)	0.60	0.45	0.38
Quality of Dialysis Center Care and Operations (QoC)	0.37	0.65	0.58
Providing Information to Patients (PI)	0.17	0.26	0.31

All correlations are significant at *P* < 0.05

The Shapiro-Wilk test revealed that the majority of survey items exhibited a non-normal distribution, with the exception of items Q5 Doctor shows respect, Q12 Staff show respect, Q27 Staff explain tests, Q33 On machine within 15 min, and Q39 Doctor/staff talk about peritoneal dialysis, which were normally distributed. Consequently, Spearman’s rank correlation was primarily employed for the construct validity analysis, while Pearson’s correlation was restricted to the normally distributed items.

The correlations between each multi-item scale and all three global rating scales are outlined in Table 4. Five out of six items from the NCC domain demonstrated a strong correlation with the doctor global rating scale (*r* = 0.51–0.58). Additionally, ten out of 17 items in the QoC domain were associated with the staff and center rating scales (*r* = 0.45–0.59 and *r* = 0.34–0.55, respectively). However, the PI domain exhibited no significant correlation with any of the three global rating scales. The correlation coefficients for all PI items were weak and negative (*r* = -0.32 to -0.01).

**Table 4:** Item-scale correlations of the Thai CAHPS-ICH composites

Items	Scales		
	Doctor rating	Staff rating	Center rating
Q3 Doctor listens carefully (*n* = 176)	0.51^a^	0.42 ^a^	0.38 ^a^
Q4 Doctor explains things (*n* = 175)	0.58 ^a^	0.42 ^a^	0.43 ^a^
Q5 Doctor shows respect (*n* = 173)	0.53^a^	0.36^a^	0.30^a^
Q6 Doctor spends enough time (*n* = 175)	0.54 ^a^	0.37 ^a^	0.42 ^a^
Q7 Doctor cared about you^*^ (*n* = 175)	0.55 ^a^	0.48 ^a^	0.55 ^a^
Q9 Doctor seemed informed (*n* = 163)	-0.03	-0.07	<-0.001
Q10 Staff listen carefully (*n* = 174)	0.28^a^	0.48^a^	0.37^a^
Q11 Staff explain in a way that is easy to understand (*n* = 174)	0.33^a^	0.50^a^	0.44^a^
Q12 Staff show respect (*n* = 172)	0.38^a^	0.54^a^	0.47^a^
Q13 Staff spend enough time (*n* = 173)	0.38^a^	0.51^a^	0.39^a^
Q14 Staff cared about you (*n* = 174)	0.34^a^	0.57^a^	0.42^a^
Q15 Staff makes you comfortable (*n* = 173)	0.37^a^	0.54^a^	0.46^a^
Q16 Staff keep information private (*n* = 168)	0.02	-0.08	-0.04
Q17 Comfortable asking staff (*n* = 173)	-0.08	-0.03	0.02
Q21 Staff insert needle without pain (*n* = 171)	0.07	0.16^a^	0.15
Q22 Staff check you closely (*n* = 173)	0.33^a^	0.54^a^	0.47^a^
Q24 Staff manage problems (*n* = 168)	-0.04	-0.04	-0.06
Q25 Staff professional (*n* = 170)	0.33^a^	0.47^a^	0.34^a^
Q26 Staff discuss diet (*n* = 174)	-0.24^a^	-0.25^a^	-0.14
Q27 Staff explain tests (*n* = 176)	0.32^a^	0.47^a^	0.35^a^
Q33 On machine within 15 min (*n* = 174)	0.20^a^	0.25^a^	0.29^a^
Q34 Center clean (*n* = 175)	0.33^a^	0.46^a^	0.55^a^
Q43 Satisfied with way problems handled (*n* = 167)	-0.15^a^	-0.27^a^	-0.22^a^
Q19 Know how to care of access site (*n* = 173)	-0.06	-0.20^a^	-0.21^a^
Q28 Staff give information on patient rights (*n* = 169)	-0.25^a^	-0.30^a^	-0.22^a^
Q29 Staff review patient rights (*n* = 167)	-0.30^a^	-0.31^a^	-0.32^a^
Q30 Staff told you what to do if health problem at home (*n* = 176)	-0.13	-0.24^a^	-0.15^a^
Q31 Staff told you how to get off machine if emergency (*n* = 173)	-0.03	-0.11	-0.15^a^
Q36 Doctor/staff talk about which treatment is right for you (*n* = 165)	-0.10	-0.04	-0.06
Q38 Doctor/staff explain why not eligible for transplant (*n* = 171)	-0.14	-0.14	-0.13
Q39 Doctor/staff talk about peritoneal dialysis (*n* = 168)	-0.16^a^	-0.18^a^	-0.16^a^
Q40 Involved in choosing treatment (*n* = 168)	-0.08	-0.07	-0.01

^a^*p* < 0.05

Values for Q5, Q12, Q27, Q33, Q37, and Q39 represent Pearson’s correlation coefficients

Values for all other items represent Spearman’s rank correlation coefficients

### The psychometric properties of Thai CAHPS-ICH

The internal consistency reliability and CFA results are presented in Tables 5 and 6, respectively.

The NCC scale demonstrated good internal consistency reliability (Cronbach’s α = 0.86). The CFA model for the NCC domain demonstrated a poor to acceptable fit (RMSEA, 0.137; CFI, 0.945; SRMR, 0.041). Five of the six factor loadings were high, ranging from 0.79 to 0.86. In contrast, the factor loading for item Q9 (Doctor seemed informed) was low and negative (-0.07).

The QoC scale exhibited acceptable internal consistency reliability (α = 0.78). The CFA model demonstrated an acceptable fit (RMSEA, 0.098; CFI, 0.844; SRMR, 0.071). Ten of the seventeen factor loadings were high, ranging from 0.57 to 0.87.

The PI scale did not demonstrate acceptable internal consistency (α = 0.53). However, the CFA model showed an acceptable fit (RMSEA, 0.066; CFI, 0.843; SRMR, 0.070). The factor loadings ranged from 0.17 to 0.66. To further investigate this, a detailed item-level analysis was performed (Supplementary Appendix Table S7). The analysis revealed that several items, particularly Q38, Q30, and Q19, had corrected item-total correlations below the 0.2 threshold (0.14, 0.17, and 0.13, respectively). Notably, the ‘Cronbach’s alpha if item deleted’ analysis showed that removing item Q38 would increase the domain’s alpha to 0.59, suggesting it was the primary item attenuating the domain’s reliability.

**Table 5:** Internal consistency reliability

Domains	Cronbach’s alpha (α)
Nephrologists’ Communication and Caring (6 items)	0.86
Quality of Dialysis Center Care and Operations (17 items)	0.78
Providing Information to Patients (9 items)	0.53

**Table 6:** Confirmatory factor analysis of the hypothesized three-composite structure

Item	Standardized Factor Loadings	95% Confidence Interval	*P* value
**Nephrologists’ communication and caring (n = 166)** ^**a**^			
Q3 Doctor listens carefully	0.79	0.72, 0.85	< 0.0001
Q4 Doctor explains things	0.85	0.80, 0.91	< 0.0001
Q5 Doctor shows respect	0.79	0.72, 0.86	< 0.0001
Q6 Doctor spends enough time	0.78	0.71, 0.85	< 0.0001
Q7 Doctor cared about you	0.81	0.74, 0.87	< 0.0001
Q9 Doctor seemed informed	-0.07	-0.23, 0.09	0.376
**Quality of Dialysis Center Care and Operations (n = 142)** ^**b**^			
Q10 Staff listen carefully	0.75	0.67, 0.83	< 0.0001
Q11 Staff explain in a way that is easy to understand	0.79	0.71, 0.85	< 0.0001
Q12 Staff show respect	0.86	0.81, 0.91	< 0.0001
Q13 Staff spend enough time	0.81	0.75, 0.87	< 0.0001
Q14 Staff cared about you	0.87	0.82, 0.92	< 0.0001
Q15 Staff makes you comfortable	0.82	0.76, 0.88	< 0.0001
Q16 Staff keep information private	-0.26	-0.42, -0.11	0.001
Q17 Comfortable asking staff	-0.14	-0.31, 0.02	0.091
Q21 Staff insert needle without pain	0.10	-0.06, 0.27	0.225
Q22 Staff check you closely	0.69	0.60, 0.79	< 0.0001
Q24 Staff manage problems	0.01	-0.16, 0.18	0.893
Q25 Staff professional	0.63	0.53, 0.74	< 0.0001
Q26 Staff discuss diet	-0.27	-0.43, -0.12	0.001
Q27 Staff explain tests	0.54	0.42, 0.67	< 0.0001
Q33 On machine within 15 min	0.31	0.16, 0.46	< 0.0001
Q34 Center clean	0.57	0.45, 0.69	< 0.0001
Q43 Satisfied with way problems handled	-0.28	-0.43, -0.12	< 0.0001
**Providing Information to Patients (n = 150)** ^**c**^			
Q19 Know how to care of access site	0.33	0.15, 0.51	< 0.0001
Q28 Staff give information on patient rights	0.59	0.43, 0.75	< 0.0001
Q29 Staff review patient rights	0.66	0.51, 0.81	< 0.0001
Q30 Staff told you what to do if health problem at home	0.21	0.02, 0.39	0.029
Q31 Staff told you how to get off machine if emergency	0.60	0.44, 0.76	< 0.0001
Q36 Doctor/staff talk about which treatment is right for you	0.17	-0.01, 0.37	0.070
Q38 Doctor/staff explain why not eligible for transplant	0.17	-0.02, 0.36	0.077
Q39 Doctor/staff talk about peritoneal dialysis	0.36	0.19, 0.54	< 0.0001
Q40 Involved in choosing treatment	0.25	0.06, 0.45	0.009

^a^Likelihood ratio test of model versus saturated: Root mean squared error of approximation (RMSEA), 90% CI = 0.137 (0.093, 0.185); comparative fit index (CFI) = 0.945; standardized root mean squared residual (SRMR) = 0.041

^b^Likelihood ratio test of model versus saturated: RMSEA, 90% CI = 0.098 (0.083, 0.113); CFI = 0.844; SRMR = 0.071

^c^Likelihood ratio test of model versus saturated: RMSEA, 90% CI = 0.066 (0.027, 0.099); CFI = 0.843; SRMR = 0.070

Most of the multi-item scales exhibited test-retest repeatability (intraclass correlation coefficient; ICC, 0.55–0.85). However, one multi-item scale (Q43 Satisfied with way problems handled) did not demonstrate this reliability (ICC = 0.19). Test-retest reliability was observed for 4 out of the 13 yes/no questions, all of which are in the PI domain (Q29, Q30, Q31, and Q38) as shown in Table 7.

**Table 7:** Intraclass correlation

Items	ICC	95%CI	*P* value
Q3 Doctor listens carefully	0.66	0.54–0.75	< 0.001
Q4 Doctor explains things	0.68	0.67–0.77	< 0.001
Q5 Doctor shows respect	0.66	0.53–0.75	< 0.001
Q6 Doctor spends enough time	0.59	0.43–0.70	< 0.001
Q7 Doctor cared about you	0.56	0.40–0.68	< 0.001
Q8 Nephrologists’ communication and caring^2^	0.64	0.51–0.73	< 0.001
Q9 Doctor seemed informed^1^	0.48	0.28–0.62	< 0.001
Q10 Staff listen carefully	0.70	0.59–0.78	< 0.001
Q11 Staff explain in a way that is easy to understand	0.65	0.52–0.74	< 0.001
Q12 Staff show respect	0.70	0.60–0.78	< 0.001
Q13 Staff spend enough time	0.66	0.53–0.75	< 0.001
Q14 Staff cared about you	0.62	0.49–0.72	< 0.001
Q15 Staff makes you comfortable	0.63	0.49–0.72	< 0.001
Q16 Staff keep information private^1^	0.22	-0.07–0.43	0.061
Q17 Comfortable asking staff^1^	-0.06	-0.44–0.22	0.638
Q21 Staff insert needle without pain	0.85	0.79–0.89	< 0.001
Q22 Staff check you closely	0.57	0.42–0.68	< 0.001
Q24 Staff manage problems	0.50	0.31–0.63	< 0.001
Q25 Staff professional	0.61	0.47–0.72	< 0.001
Q26 Staff discuss diet^1^	0.30	0.05–0.49	0.012
Q27 Staff explain tests	0.61	0.47–0.71	< 0.001
Q32 Quality of Dialysis Center Care and Operations^2^	0.70	0.60–0.78	< 0.001
Q33 On machine within 15 min	0.65	0.52–0.74	< 0.001
Q34 Center clean	0.54	0.37–0.66	< 0.001
Q35 Providing Information to Patients^2^	0.79	0.72–0.85	< 0.001
Q43 Satisfied with way problems handled	0.19	-0.12–0.41	0.105
Q19 Know how to care of access site^1^	0.28	0.02–0.47	0.020
Q28 Staff give information on patient rights^1^	0.13	-0.19–0.37	0.190
Q29 Staff review patient rights^1^	0.68	0.55–0.77	< 0.001
Q30 Staff told you what to do if health problem at home^1^	0.58	0.42–0.69	< 0.001
Q31 Staff told you how to get off machine if emergency^1^	0.57	0.41–0.69	< 0.001
Q36 Doctor/staff talk about which treatment is right for you^1^	0.34	0.09–0.52	0.006
Q38 Doctor/staff explain why not eligible for transplant^1^	0.70	0.59–0.79	< 0.001
Q39 Doctor/staff talk about peritoneal dialysis^1^	0.47	0.27–0.61	< 0.001
Q40 Involved in choosing treatment^1^	0.44	0.23–0.59	< 0.001

^1^Yes/No question

^2^Global rating scale

We compared the results of our “Thai CAHPS-ICH” survey and the original ICH-CAHPS^®^ survey in Table 8. The response rate in our study was higher than that of the original survey (80.43% vs. 46%). Both surveys demonstrated internal consistency in the NCC and QoC domains (⍺ = 0.86 and 0.78 in the Thai CAHPS-ICH survey, and ⍺ = 0.81 and 0.90 in the ICH-CAHPS^®^ survey, respectively). However, neither survey showed internal consistency in the PI domain (⍺ = 0.53 in the Thai CAHPS-ICH survey and ⍺ = 0.55 in the ICH-CAHPS^®^ survey). Regarding validity, both surveys exhibited correlations between the NCC domain and the doctor rating scale (*r* = 0.60 and 0.78 in the Thai CAHPS-ICH and original ICH-CAHPS^®^ surveys, respectively), between the QoC domain and the staff rating scale (*r* = 0.65 and 0.75 in the Thai CAHPS-ICH and original ICH-CAHPS^®^ surveys, respectively), and between the QoC and the center rating scale (*r* = 0.58 and 0.69 in the Thai CAHPS-ICH and original ICH-CAHPS^®^ surveys, respectively). However, both surveys showed less strong correlations between the PI domain and all global rating scales.

**Table 8:** Comparison between the Thai CAHPS-ICH and original ICH-CAHPS^®^(6, 22)

Contents	Thai CAHPS-ICH	Original ICH-CAHPS^®^
Study design (n)	Single center study in Chiang Mai (*n* = 189)	Multicenter study in US (*n* = 819)
Data collection	Questionnaire form	Telephone or mailed survey
Response rate	80.43%	46%
Reliability	⍺^1^ = 0.86 (NCC), 0.78 (QoC), 0.53 (PI)	⍺^1^ = 0.81 (NCC), 0.90 (QoC), 0.55 (PI)
Validity(r)^2^	- 0.60 between NCC and Doctor GRS - 0.65 between QoC and Staff GRS - 0.58 between QoC and Center GRS - < 0.5 between PI domain and all GRS	- 0.78 between NCC and Doctor GRS - 0.75 between QoC and Staff GRS - 0.69 between QoC and Center GRS - < 0.5 between PI domain and all GRS

^1^Cronbachs’ alpha coefficient

^2^Correlation coefficient (r) of value ≥ 0.3 and ≥ 0.4 were considered association for Thai CAHPS-ICH and for Original ICH-CAHPS respectively

## Discussion

This was the first study to develop and evaluate our “Thai CAHPS-ICH” survey for MHD patients in Thailand. As the initial phase of our project, we intended to verify that the survey could project the details of the quality of care. Considering the limited existing evidence on patient experience and care quality, our subsequent objective is to explore the relationship between survey outcomes and clinical patient outcomes. Our findings suggest that the “Thai CAHPS-ICH” survey demonstrates construct validity and achieves acceptable to good internal consistency reliability.

The exclusion of 18 patients (6.1%) with a Karnofsky Performance Status (KPS) score below 50 was a methodological choice to ensure data integrity. In hemodialysis patients, low functional status is strongly associated with elevated levels of depression and anxiety [22], both of which may confound the self-reporting of patient experiences. By maintaining a higher KPS threshold, we aimed to minimize the impact of psychological distress on the survey’s internal validity, ensuring that responses reflected the actual quality of care rather than the patient’s psychological state.

The ceiling effects were significant across all three global rating scales and the majority of multi-item scales, except for Q24 and Q43. Conversely, the floor effects were minimal. These ceiling effects were consistent with those observed in the original ICH-CAHPS^®^ survey, ranging from 15% in the QoC composite to 75% in the kidney doctor global rating [6]. These findings may be attributable to the high standard of care provided by our academic hemodialysis facility, or they could be influenced by response bias among the patient population. However, we must also consider the potential influence of social desirability bias. In the absence of direct comparisons with national objective care indicators—such as standardized mortality ratios or infection rates—it remains unclear whether these high ratings are strictly a reflection of clinical excellence or are partially inflated by patients’ inclination to provide positive feedback. Consequently, the findings from this high-resource setting may not be fully representative of the experiences in rural or private hemodialysis centers where resource availability and care delivery models differ. Future multi-center research encompassing diverse healthcare settings is essential to evaluate the survey’s discriminative power and establish a broader national benchmark for Thai MHD patients.

The assessment of construct validity through CFA is critical, but the results were mixed and require careful interpretation. While the QoC and PI domain models demonstrated an acceptable fit to the data, the NCC model exhibited a poor-to-mediocre fit, suggesting that the structure of the communication domain may not translate as robustly to the Thai context as the more concrete QoC domain. The most critical issue observed in the NCC model was the low and negative factor loading for item Q9 (“Doctor seemed informed”), an anomaly that indicates the item may be poorly understood or culturally redundant in a context of high deference, thus limiting the definitive confirmation of the original three-factor structure. Notably, a sensitivity analysis revealed that removing item Q9 resulted in a further deterioration of the model fit (RMSEA = 0.205, CFI = 0.933) (Supplementary Appendix Table S5). Consequently, despite its low factor loading, item Q9 was retained in the final Thai version to maintain the instrument’s integrity and international comparability. While the CFA provided initial evidence supporting the overall structure, the mixed fit and the problematic item in the NCC domain limit the structural validity of the instrument, suggesting that the model is underspecified and requires further refinement, specifically by revising item Q9 and potentially employing Exploratory Factor Analysis (EFA) in a new sample to empirically confirm the optimal factor structure.

The internal consistency of the PI domain was limited, primarily driven by the poor performance of item Q38 (‘Staff talked about what to do if you have a health problem at home’). As shown in the item-level analysis (Supplementary Appendix Table S7), the widespread weakness across multiple items—rather than a single outlier—reinforces the argument that the binary (Yes/No) response format is insufficiently sensitive to capture the variance in Thai patients’ experiences. We retained these items to preserve the structural integrity of the original CAHPS-ICH during this initial validation phase; however, these findings highlight a critical need for revising the PI items into Likert-scale formats in future studies to improve psychometric robustness.

In assessing the construct validity of this survey, we observed large associations between the NCC domain and the kidney doctor rating scale, the QoC domain and staff rating scale, as well as the QoC domain and center rating scale. These findings indicate that the expected measure within each domain correlated with the intended aspects of assessment. The strongest correlations within each domain were similar to those found in the original survey [23]. However, the PI domain, aimed at evaluating patients’ understanding of the information provided by nephrologists or hemodialysis staff, did not exhibit a significant correlation with any global rating scale. This negative result might be because none of the three global rating scales were specifically designed to assess this aspect.

In evaluating the internal consistency and test-retest reliabilities of the Thai CAHPS-ICH survey, we found that the NCC and QoC domains exhibited good and acceptable internal consistency reliability, respectively. These results correspond with those of the original ICH-CAHPS^®^ survey in the United States, suggesting that the core constructs related to staff and physician interaction translate robustly across different healthcare systems and cultures [6, 23]. However, similar to the original ICH-CAHPS^®^ survey, the PI domain did not demonstrate acceptable internal consistency [6]. This issue may stem from several factors. Potential reasons for this outcome could include the heterogeneous nature of the questions within this domain and variations in patients’ levels of literacy. More critically, the PI items are primarily binary (Yes/No) questions, which inherently limit score variance and make achieving high internal consistency difficult compared to multi-point Likert scales. Furthermore, variations in patients’ levels of health literacy or understanding of abstract concepts like ‘shared decision-making’ may contribute to the unreliable responses in this specific domain. Therefore, while the PI domain measures an important construct, researchers utilizing this tool must treat this domain with caution. To enhance the reliability and statistical utility of this domain in future adaptations, we recommend converting the binary PI items to a multi-point Likert scale.

Cultural disparities in the patient-provider relationship may have introduced a response bias that influenced the Thai CAHPS-ICH scores compared to Western contexts. According to Hofstede’s cultural dimensions, Thai culture exhibits high power distance, often manifesting as ‘Kreng-jai’—a pervasive social norm of deference and reluctance to criticize authority figures such as physicians [24]. This cultural backdrop suggests that the consistently high scores in the NCC domain may partially reflect a ‘cultural halo effect,’ where patients prioritize emotional care, politeness, and social harmony over a strictly objective assessment of technical communication quality. Consequently, high satisfaction ratings might be driven by staff behavior and professionalism rather than the actual effectiveness of information exchange. To disentangle this cultural deference from true quality of care, future research should investigate the correlation between NCC scores and more nuanced objective outcomes appropriate for the Thai clinical context. Rather than simple time-based metrics, we suggest focusing on patient-held knowledge post-consultation (e.g., accurate understanding of treatment goals) or treatment adherence indicators (e.g., consistency in interdialytic weight gain control). Testing whether high NCC scores correlate with better patient understanding or improved clinical compliance would provide robust evidence of whether the instrument reflects effective clinical communication or merely a cultural inclination toward respect for authority.

Our study had several strengths. First, our survey represented the first Patient-Reported Experience Measure for maintenance hemodialysis patients in Thailand, and we expected that this survey could be used to assess the quality of care among hemodialysis centers in Thailand. The validation of the Thai CAHPS-ICH provides a standardized, patient-centered metric that allows nephrologists and healthcare administrators to move beyond traditional biochemical markers, such as Kt/V or hemoglobin levels, to capture the lived experiences of patients. Second, we recruited all patients from a single hemodialysis center, which confirmed that the results apparently reflected the quality of care provided by the individual hemodialysis facility.

However, some limitations must be acknowledged. First, because of the absence of validated PREMs for Thai MHD patients, the comparison of each domain with its own components was not directly validated. To address these issues, future research must prioritize evaluating criterion validity by linking survey scores to prospective clinical outcomes. Building on international evidence, our planned longitudinal follow-up study (Phase 2) will empirically examine the survey’s predictive utility regarding clinical parameter such as interdialytic weight gain, emergency department visits, and mortality within the Thai population [25]. Second, we did not evaluate the discriminant validity of the survey, which is necessary to confirm that each domain measures a unique construct. This omission may limit the utility of the results for comparative quality assessments across different domains. Third, this was a single-center study that could not be generalized to other hemodialysis centers in Thailand. Therefore, multi-center studies involving diverse settings—such as private units and dialysis centers in government hospitals—are necessary to enhance generalizability. Finally, while the validation protocol followed the original CAHPS-ICH standards to facilitate cross-national comparability, it did not fully adopt the COSMIN checklist for all psychometric reporting. Future validation studies of the Thai CAHPS-ICH should integrate COSMIN-recommended standards, including the calculation of content validity indices and responsiveness to change, to further align the instrument with global psychometric standards.

This study successfully confirmed the preliminary psychometric properties of the Thai CAHPS-ICH survey within a tertiary care hemodialysis center. However, key issues require further investigation to fully establish its utility in the Thai context. First, generalizability must be evaluated through subsequent studies that collect data from a broader and more diverse sample of patients across Thailand. This national-level implementation should include stand-alone dialysis clinics, public hospitals, and private hospitals to confirm the stability of the factor structure across various healthcare settings. Second, prospective studies are needed to investigate the relationship between the survey scores and critical patient clinical outcomes (e.g., laboratory parameters, health-related quality of life [HRQoL], treatment adherence, hospitalization rates, and mortality). Establishing these correlations will be crucial for confirming the criterion validity of the survey in practical clinical use. Third, in the long term, research should focus on planning the implementation of the Thai CAHPS-ICH as a standardized performance measurement tool for assessing the quality of patient care nationwide, thus promoting systemic quality improvement and benchmarking among hemodialysis centers throughout Thailand.

## Conclusion

The Thai version of the Consumer Assessment of Health Care Providers and Systems In-Center Hemodialysis, or “Thai CAHPS-ICH” survey, demonstrates reliability and validity among patients undergoing maintenance hemodialysis at Maharaj Nakorn Chiang Mai Hospital. This survey holds potential for application in other hemodialysis facilities across Thailand.

## Electronic Supplementary Material

Below is the link to the electronic supplementary material.

Supplementary Material 1

## Data Availability

The datasets used and/or analyzed during the current study available from the corresponding author on reasonable request.

## Abbreviations

AHRQ: Agency for Healthcare Research and Quality
CFA: Confirmatory Factor Analysis
CFI: Comparative Fit Index
CMS: Centers for Medicare & Medicaid Services
ESKD: End-Stage Kidney Disease
ESRD: End-Stage Renal Disease
ESRD QIP: End-Stage Renal Disease Quality Incentive Program
HRQoL: Health-Related Quality of Life
ICC: Intraclass Correlation Coefficient
IRB: Institutional Review Board
MHD: Maintenance Hemodialysis
NCC: Nephrologists’ Communication and Caring
PI: Providing Information to Patients
PREMs: Patient-Reported Experience Measures
PROMs: Patient-Reported Outcome Measures
QoC: Quality of Dialysis Center Care and Operation
RMSEA: Root Mean Square Error of Approximation
RRT: Renal Replacement Therapy
SRMR: Standardized Root Mean Square Residual
Thai CAHPS-ICH: Thai Version Consumer Assessment of Healthcare Providers and Systems In-Centre Hemodialysis Survey
USRDS: United States Renal Data System
ICH-GCP: International Conference on Harmonisation - Good Clinical Practice
SOP: Standard Operating Procedures
WHO: World Health Organization
