# Supplementary material for: Validity and reliability of the Thai version Consumer Assessment of Healthcare Providers and Systems In-Centre Hemodialysis (Thai CAHPS-ICH) survey for maintenance hemodialysis patients
Source: BMC Nephrol. 2026 Jun 4;27:465. doi: 10.1186/s12882-026-05042-5 (PMC13459310; doi:10.1186/s12882-026-05042-5)
Supplement: Supplementary file 1 [file 12882_2026_5042_MOESM1_ESM.docx]

**Supplementary Appendix**

**This contains**

1. **Translated version of Thai CAHPS-ICH survey**
2. **Thai CAHPS-ICH survey (provided in Thai language)**
3. **Figure S1,S2**
4. **Table S1 to S8**

**Survey for Service Users on Staff and Service System for EndStage Renal Disease Patients Receiving Hemodialysis (Thai Consumer Assessment of Healthcare Providers and Systems – InCenter Hemodialysis, Thai CAHPSICH)**

**How to complete this survey**

Please put a 🗸 in the 🞎 box. In case this is done as an interview, the patient/recipient of care should answer the questions truthfully.

In some questions, after you answer, you may be instructed to skip to another question. In those cases, you will see directions such as:

🞎 Yes 🡪 **If you answer this, skip to Question 1**

🞎 No

1. Where do you receive your dialysis?

🞎^1^ At home 🡪 **If you answer this, stop filling out the questionnaire.**

🞎^2^ At a hemodialysis center

2. How long have you been receiving dialysis at this center?

🞎^1^ Less than 3 months 🡪 **If you answer this, stop filling out the questionnaire.**

🞎^2^ 3 months to less than 1 year

🞎^3^ 1 year to less than 5 years

🞎^4^ 5 years or more

Section 1: Your kidney doctor (nephrologist)

For the following questions, “your kidney doctor” means the doctor or group of doctors who manage your dialysis treatment at this dialysis center.

3. In the last 3 months, how often did your kidney doctor listen carefully to you?

🞎^1^ Never

🞎^2^ Sometimes

🞎^3^ Often

🞎^4^ Always

4. In the last 3 months, how often did your kidney doctor explain things in a way that was easy for you to understand?

🞎^1^ Never

🞎^2^ Sometimes

🞎^3^ Often

🞎^4^ Always

5. In the last 3 months, how often did your kidney doctor give importance to what you had to say?

🞎^1^ Never

🞎^2^ Sometimes

🞎^3^ Often

🞎^4^ Always

6. In the last 3 months, how often did your kidney doctor spend enough time with you?

🞎^1^ Never

🞎^2^ Sometimes

🞎^3^ Often

🞎^4^ Always

7. In the last 3 months, how often did you feel that your kidney doctor genuinely cared about you?

🞎^1^ Never

🞎^2^ Sometimes

🞎^3^ Often

🞎^4^ Always

8. What number from 0 to 10 would you use to rate your kidney doctor?
   (0 means the worst doctor possible, and 10 means the best doctor possible.)

🞎 0 Worst

🞎 1

🞎 2

🞎 3

🞎 4

🞎 5

🞎 6

🞎 7

🞎 8

🞎 9

🞎 10 Best

9. Does your kidney doctor know about the treatments you receive from other doctors?

🞎^1^ Yes

🞎^2^ No

Section 2 Staff at the dialysis center

For the following questions, “staff at the dialysis center” includes nurses, technicians, dietitians, and social workers at this dialysis center. It does **not include** doctors.

10. In the last 3 months, how often did the dialysis center staff listen carefully to you?

🞎^1^ Never

🞎^2^ Sometimes

🞎^3^ Often

🞎^4^ Always

11. In the last 3 months, how often did the dialysis center staff explain things in a way that was easy for you to understand?

🞎^1^ Never

🞎^2^ Sometimes

🞎^3^ Often

🞎^4^ Always

12. In the last 3 months, how often did the dialysis center staff give importance to what you had to say?

🞎^1^ Never

🞎^2^ Sometimes

🞎^3^ Often

🞎^4^ Always

13. In the last 3 months, how often did the dialysis center staff spend enough time with you?

🞎^1^ Never

🞎^2^ Sometimes

🞎^3^ Often

🞎^4^ Always

14. In the last 3 months, how often did you feel that the dialysis center staff paid attention to and cared about you?

🞎^1^ Never

🞎^2^ Sometimes

🞎^3^ Often

🞎^4^ Always

15. In the last 3 months, how often did the dialysis center staff make you feel comfortable during dialysis?

🞎^1^ Never

🞎^2^ Sometimes

🞎^3^ Often

🞎^4^ Always

16. In the last 3 months, did the dialysis center staff keep your personal information and health information strictly confidential from other patients?

🞎^1^ Yes

🞎^2^ No

17. In the last 3 months, did you feel comfortable asking the dialysis center staff any questions you had about dialysis?

🞎^1^ Yes

🞎^2^ No

18. In the last 3 months, did any dialysis center staff ask you about how kidney disease affects your daily life?

🞎^1^ Yes

🞎^2^ No

19. Do you know how to take care of your dialysis access site?

🞎^1^ Yes

🞎^2^ No

20. In the last 3 months, which type of access did the dialysis center staff use for your dialysis?

🞎^1^ Artificial blood vessel in the arm (graft)

🞎^2^ Natural blood vessel in the arm (fistula)

🞎^3^ Central venous catheter 🡪 **If you answer this, skip to Question 22.**

🞎^4^ Do not know 🡪 **If you answer this, skip to Question 22.**

21. In the last 3 months, how often did the dialysis center staff insert the needles gently?

🞎^1^ Never

🞎^2^ Sometimes

🞎^3^ Often

🞎^4^ Always

🞎^5^ I insert the needles myself

22. In the last 3 months, during dialysis, how often did the dialysis center staff check on you as closely as you wanted?

🞎^1^ Never

🞎^2^ Sometimes

🞎^3^ Often

🞎^4^ Always

23. In the last 3 months, did any problems occur during your dialysis treatment?

🞎^1^ Yes

🞎^2^ No 🡪 **If you answer this, skip to Question 25.**

24. In the last 3 months, how often were the dialysis center staff able to resolve the problems that occurred during your dialysis treatment?

🞎^1^ Never

🞎^2^ Sometimes

🞎^3^ Often

🞎^4^ Always

25. In the last 3 months, how often did the dialysis center staff act in a professional manner?

🞎^1^ Never

🞎^2^ Sometimes

🞎^3^ Often

🞎^4^ Always

26. In the last 3 months, did the dialysis center staff talk with you about what foods and drinks you should have?

🞎^1^ Yes

🞎^2^ No

27. In the last 3 months, how often did the dialysis center staff explain your blood test results in a way that was easy for you to understand?

🞎^1^ Never

🞎^2^ Sometimes

🞎^3^ Often

🞎^4^ Always

28. Patients have various rights, such as the right to be treated with respect and the right to privacy as a patient. Have the dialysis center staff ever shown you a consent form that explains the rights you are entitled to, and asked you to sign to acknowledge that you have been informed?

🞎^1^ Yes

🞎^2^ No

29. Have the dialysis center staff ever reviewed your patient rights with you?

🞎^1^ Yes

🞎^2^ No

30. Have the dialysis center staff ever told you what to do if you have a health problem while you are at home?

🞎^1^ Yes

🞎^2^ No

31. Have the dialysis center staff ever told you how to get off the dialysis machine in case of an emergency at the center?

🞎^1^ Yes

🞎^2^ No

32. What number from 0 to 10 would you use to rate the dialysis center staff?
    (0 means the worst staff possible, and 10 means the best staff possible.)

🞎 0 Worst

🞎 1

🞎 2

🞎 3

🞎 4

🞎 5

🞎 6

🞎 7

🞎 8

🞎 9

🞎 10 Best

Section 3 The hemodialysis center

33. In the last 3 months, when you arrived at the dialysis center on time for your appointment, how often did you get on the dialysis machine within 15 minutes?

🞎^1^ Never

🞎^2^ Sometimes

🞎^3^ Often

🞎^4^ Always

34. In the last 3 months, how often did you feel that the dialysis center was kept clean overall?

🞎^1^ Never

🞎^2^ Sometimes

🞎^3^ Often

🞎^4^ Always

35. What number from 0 to 10 would you use to rate the dialysis center?
    (0 means the worst center possible, and 10 means the best center possible.)

🞎 0 Worst

🞎 1

🞎 2

🞎 3

🞎 4

🞎 5

🞎 6

🞎 7

🞎 8

🞎 9

🞎 10 Best

Section 4 Medical care

The following questions are about the medical care you received during the past 12 months.

36. Patients with kidney disease can be treated with a kidney transplant, by receiving hemodialysis at a center, or by peritoneal dialysis. In the last 12 months, did your kidney doctor or the dialysis center staff give you as much information as you wanted about the treatment options that are suitable for you?

🞎^1^ Yes

🞎^2^ No

37. Are you eligible for a kidney transplant?

🞎^1^ Yes 🡪 **If you answer this, skip to Question 39**

🞎^2^ No

🞎^3^ Do not know 🡪 **If you answer this, skip to Question 39**

38. In the last 12 months, did your kidney doctor or the dialysis center staff explain the reasons why you could not receive a kidney transplant?

🞎^1^ Yes

🞎^2^ No

39. In the last 12 months, did your kidney doctor or the dialysis center staff talk with you about peritoneal dialysis?

🞎^1^ Yes

🞎^2^ No

40. In the last 12 months, were you involved in choosing the kidney treatment that was appropriate for you?

🞎^1^ Yes

🞎^2^ No

41. In the last 12 months, have you ever felt dissatisfied with the care you received from the dialysis center or from your kidney doctor?

🞎^1^ Yes

🞎^2^ No 🡪 **If you answer this, skip to Question ข้อ 45**

42. **From Question 41**: In the last 12 months, did you ever talk with anyone else about your dissatisfaction with the dialysis center staff?

🞎^1^ Yes

🞎^2^ No 🡪 **If you answer this, skip to Question 45**

43. In the last 12 months, how often were you satisfied with the way the hemodialysis center staff handled problems related to your treatment?

🞎^1^ Never

🞎^2^ Sometimes

🞎^3^ Often

🞎^4^ Always

44. The Provincial Public Health Office where you live can inspect and monitor the quality of services at dialysis centers. In the last 12 months, have you ever made a complaint to this office?

🞎^1^ Yes

🞎^2^ No 🡪 **If you answer this, skip to Question 45**

Section 5: About you

45. How would you rate your overall health?

🞎^1^ Excellent

🞎^2^ Very good

🞎^3^ Good

🞎^4^ Fair

🞎^5^ Poor

46. How would you rate your overall mental health and emotional wellbeing?

🞎^1^ Excellent

🞎^2^ Very good

🞎^3^ Good

🞎^4^ Fair

🞎^5^ Poor

47. Are you currently being treated for high blood pressure?

🞎^1^ Yes

🞎^2^ No

48. Are you currently being treated for diabetes or high blood sugar?

🞎^1^ Yes

🞎^2^ No

49. Are you currently being treated for heart disease or a heartrelated condition?

🞎^1^ Yes

🞎^2^ No

50. Please select your age group:

🞎^1^ 18 - 24 years

🞎^2^ 25 - 34 years

🞎^3^ 35 - 44 years

🞎^4^ 45 - 54 years

🞎^5^ 55 - 64 years

🞎^6^ 65 - 74 years

🞎^7^ 75 years or older

51. Please select your sex:

🞎^1^ Male

🞎^2^ Female

52. Please select your highest level of education:

🞎^1^ Elementary school

🞎^2^ Junior high school

🞎^3^ Senior high school

🞎^4^ Bachelor’s degree

🞎^5^ Higher than bachelor’s degree

53. Are you a foreign national (nonThai citizen)?

🞎^1^ Yes

🞎^2^ No

54. Please select your ethnicity:

🞎^1^ Thai

🞎^2^ Other

55. What language do you mainly use to communicate with other people at home?

🞎^1^ Thai

🞎^2^ Thai and another language equally

🞎^3^ Other, please specify ________________________

56. Did anyone help you complete this survey?

🞎^1^ Yes

🞎^2^ No 🡪 **If you answer this, stop here.**

57. Who helped you complete this survey?

🞎^1^ Family member

🞎^2^ Friend

🞎^3^ Dialysis center staff

🞎^4^ Other, please specify ________________________

58. How did the person in Question 57 help you? (You may choose more than one.)

🞎^1^ Read the question to you

🞎^2^ Wrote down the answers for you

🞎^3^ Answered the questions for you

🞎^4^ Translated the questions into your language

🞎^5^ Other, please specify________________________

Thank you very much for your cooperation.

**แบบสำรวจสำหรับผู้รับบริการจากเจ้าหน้าที่และระบบให้บริการสำหรับผู้ป่วยโรคไตวายเรื้อรังระยะสุดท้ายที่ได้รับการฟอกเลือด(Thai consumer assessment of healthcare providers and systems in-centre hemodialysis, Thai CAHPS-ICH)**

วิธีตอบแบบสำรวจ

โปรดทำเครื่องหมาย 🗸 ในช่อง 🞎 ในกรณีที่เป็นการสัมภาษณ์ ให้ผู้ป่วย/ผู้รับการรักษาตอบคำถามให้ตรงกับความจริง

ในบางกรณีเมื่อตอบคำถามแล้ว ท่านอาจต้องข้ามไปทำข้ออื่น ซึ่งจะมีข้อความปรากฏดังนี้

🞎	ใช่ 🡪 **หากตอบข้อนี้ ให้ข้ามไปข้อ 1**

🞎	ไม่

1. ท่านรับการฟอกไตจากที่ใด

🞎^1^ ที่บ้าน 🡪 **หากตอบข้อนี้ ให้หยุดทำแบบสอบถาม**

🞎^2^ ที่ศูนย์ฟอกไต

2. ท่านใดรับการฟอกไตจากศูนย์แห่งนี้มานานเท่าใด

🞎^1^ น้อยกว่า 3 เดือน 🡪 **หากตอบข้อนี้ ให้หยุดทำแบบสอบถาม**

🞎^2^ 3 เดือน ถึง น้อยกว่า 1 ปี

🞎^3^ 1 ปี ถึง น้อยกว่า 5 ปี

🞎^4^ ตั้งแต่ 5 ปี ขึ้นไป

หมวดที่ 1 แพทย์โรคไตของท่าน

สำหรับคำถามต่อไปนี้ “แพทย์โรคไตของท่าน” หมายถึง แพทย์หรือกลุ่มแพทย์ที่ดูแลการฟอกไต ณ ศูนย์ฟอกไตแห่งนี้

3. ใน 3 เดือนที่ผ่านมา แพทย์โรคไตของท่านตั้งใจรับฟังท่านบ่อยเพียงใด

🞎^1^ ไม่เคย

🞎^2^ บางครั้ง

🞎^3^ บ่อย

🞎^4^ ประจำ

4. ในช่วง 3 เดือนที่ผ่ามา แพทย์โรคไตของท่านได้อธิบายเรื่องต่าง ๆ ให้ท่านเข้าใจได้ง่าย บ่อยเพียงใด

🞎^1^ ไม่เคย

🞎^2^ บางครั้ง

🞎^3^ บ่อย

🞎^4^ ประจำ

5. ในช่วง 3 เดือนที่ผ่านมา แพทย์โรคไตของท่านให้ความสำคัญกับสิ่งที่ท่านพูดบ่อยเพียงใด

🞎^1^ ไม่เคย

🞎^2^ บางครั้ง

🞎^3^ บ่อย

🞎^4^ ประจำ

6. ในช่วง 3 เดือนที่ผ่านมา แพทย์โรคไตของท่านได้ใช้เวลาดูแลท่านอย่างเพียงพอบ่อยเพียงใด

🞎^1^ ไม่เคย

🞎^2^ บางครั้ง

🞎^3^ บ่อย

🞎^4^ ประจำ

7. ในช่วง 3 เดือนที่ผ่านมา ท่านรู้สึกว่าแพทย์โรคไตของท่านใส่ใจท่านอย่างแท้จริงบ่อยเพียงใด

🞎^1^ ไม่เคย

🞎^2^ บางครั้ง

🞎^3^ บ่อย

🞎^4^ ประจำ

8. ท่านจะให้คะแนนแพทย์โรคไตของท่านในระดับใด

(โดย 0 เท่ากับระดับแย่ที่สุด และ 10 เท่ากับระดับดีที่สุด)

🞎 0 แย่ที่สุด

🞎 1

🞎 2

🞎 3

🞎 4

🞎 5

🞎 6

🞎 7

🞎 8

🞎 9

🞎 10 ดีที่สุด

9. แพทย์โรคไตของท่านทราบถึงข้อมูลการรักษาที่ท่านได้รับจากแพทย์ท่านอื่นหรือไม่

🞎^1^ ใช่

🞎^2^ ไม่ใช่

หมวดที่ 2 เจ้าหน้าที่ศูนย์ฟอกเลือด

สำหรับคำถามต่อไปนี้ เจ้าหน้าที่ศูนย์ฟอกเลือดได้แก่ พยาบาล นักเทคนิค นักโภชนาการ และนักสังคมสงเคราะห์ที่ศูนย์ฟอกเลือดแห่งนี้ **ไม่รวม**แพทย์

10. ใน 3 เดือนที่ผ่านมา เจ้าหน้าที่ศูนย์ฟอกเลือดตั้งใจรับฟังท่านบ่อยเพียงใด

🞎^1^ ไม่เคย

🞎^2^ บางครั้ง

🞎^3^ บ่อย

🞎^4^ ประจำ

11. ในช่วง 3 เดือนที่ผ่านมา เจ้าหน้าที่ศูนย์ฟอกเลือดได้อธิบายเรื่องต่างๆ ให้ท่านเข้าใจได้โดยง่ายบ่อยเพียงใด

🞎^1^ ไม่เคย

🞎^2^ บางครั้ง

🞎^3^ บ่อย

🞎^4^ ประจำ

12. ในช่วง 3 เดือนที่ผ่านมา เจ้าหน้าที่ศูนย์ฟอกเลือดให้ความสำคัญกับสิ่งที่ท่านพูดบ่อยเพียงใด

🞎^1^ ไม่เคย

🞎^2^ บางครั้ง

🞎^3^ บ่อย

🞎^4^ ประจำ

13. ในช่วง 3 เดือนที่ผ่านมา เจ้าหน้าที่ศูนย์ฟอกเลือดของท่านได้ใช้เวลากับท่านอย่างเพียงพอบ่อยเพียงใด

🞎^1^ ไม่เคย

🞎^2^ บางครั้ง

🞎^3^ บ่อย

🞎^4^ ประจำ

14. ในช่วง 3 เดือนที่ผ่านมา ท่านรู้สึกว่าเจ้าหน้าที่ศูนย์ฟอกเลือดได้ให้ความใส่ใจท่านบ่อยเพียงใด

🞎^1^ ไม่เคย

🞎^2^ บางครั้ง

🞎^3^ บ่อย

🞎^4^ ประจำ

15. ในช่วง 3 เดือนที่ผ่านมา เจ้าหน้าที่ศูนย์ฟอกเลือดทำให้ท่านรู้สึกสบายใจระหว่างการฟอกเลือดบ่อยเพียงใด

🞎^1^ ไม่เคย

🞎^2^ บางครั้ง

🞎^3^ บ่อย

🞎^4^ ประจำ

16. ในช่วง 3 เดือนที่ผ่านมา เจ้าหน้าที่ศูนย์ฟอกเลือดได้เก็บรักษาความลับข้อมูลส่วนตัว และข้อมูลด้านสุขภาพของท่านจากคนไข้คนอื่นอย่างรัดกุมที่สุด ใช่หรือไม่

🞎^1^ ใช่

🞎^2^ ไม่ใช่

17. ในช่วง 3 เดือนที่ผ่านมา ท่านรู้สึกสบายใจที่จะสอบถามข้อมูลทุกอย่าง เกี่ยวกับการฟอกเลือดจากเจ้าหน้าที่ศูนย์ฟอกเลือดหรือไม่

🞎^1^ ใช่

🞎^2^ ไม่ใช่

18. ในช่วง 3 เดือนที่ผ่านมา เคยมีเจ้าหน้าที่ศูนย์ฟอกเลือดสอบถามท่านถึงผลกระทบของโรคไตต่อการใช้ชีวิตของท่านหรือไม่

🞎^1^ ใช่

🞎^2^ ไม่ใช่

19. ท่านทราบวิธีดูแลเส้นฟอกเลือดของท่านหรือไม่

🞎^1^ ใช่

🞎^2^ ไม่ใช่

20. ในช่วง 3 เดือนที่ผ่านมา เจ้าหน้าที่ศูนย์ฟอกเลือดใช้เส้นใดในการฟอกเลือดของท่าน

🞎^1^ เส้นเลือดเทียมที่แขน (Graft)

🞎^2^ เส้นเลือดจริงที่แขน (Fistula)

🞎^3^ สายสวนหลอดเลือด (Catheter) 🡪 **หากตอบข้อนี้ ให้ข้ามไปข้อ 22**

🞎^4^ ไม่ทราบ 🡪 **หากตอบข้อนี้ ให้ข้ามไปข้อ 22**

21. ในช่วง 3 เดือนที่ผ่านมา เจ้าหน้าที่ศูนย์ฟอกเลือดได้แทงเข็มด้วยความเบามือบ่อยเพียงใด

🞎^1^ ไม่เคย

🞎^2^ บางครั้ง

🞎^3^ บ่อย

🞎^4^ ประจำ

🞎^5^ แทงเข็มด้วยตนเอง

22. ในช่วง 3 เดือนที่ผ่านมา ระหว่างการฟอกเลือด เจ้าหน้าที่ศูนย์ฟอกเลือดได้ดูแลท่านอย่างใกล้ชิดเท่าที่ท่านต้องการบ่อยเพียงใด

🞎^1^ ไม่เคย

🞎^2^ บางครั้ง

🞎^3^ บ่อย

🞎^4^ ประจำ

23. ในช่วง 3 เดือนที่ผ่านมา เคยมีปัญหาเกิดขึ้นระหว่างการฟอกเลือดของท่านหรือไม่

🞎^1^ ใช่

🞎^2^ ไม่ใช่ 🡪 **หากตอบข้อนี้ ให้ข้ามไปข้อ 25**

24. ในช่วง 3 เดือนที่ผ่านมา เจ้าหน้าที่ศูนย์ฟอกเลือดสามารถแก้ปัญหาที่เกิดขึ้นระหว่างการฟอกเลือดของท่านได้บ่อยเพียงใด

🞎^1^ ไม่เคย

🞎^2^ บางครั้ง

🞎^3^ บ่อย

🞎^4^ ประจำ

25. ในช่วง 3 เดือนที่ผ่านมา เจ้าหน้าที่ศูนย์ฟอกเลือดแสดงความเป็นมืออาชีพบ่อยเพียงใด

🞎^1^ ไม่เคย

🞎^2^ บางครั้ง

🞎^3^ บ่อย

🞎^4^ ประจำ

26. ใน 3 เดือนที่ผ่านมา เจ้าหน้าที่ศูนย์ฟอกเลือดได้พูดคุยกับท่านเกี่ยวกับอาหารและเครื่องดื่มที่ควรรับประทานหรือไม่

🞎^1^ ใช่

🞎^2^ ไม่ใช่

27. ในช่วง 3 เดือนที่ผ่านมา เจ้าหน้าที่ศูนย์ฟอกเลือดได้อธิบายผลเลือดให้ท่านเข้าใจโดยง่ายบ่อยเพียงใด

🞎^1^ ไม่เคย

🞎^2^ บางครั้ง

🞎^3^ บ่อย

🞎^4^ ประจำ

28. ผู้ป่วยย่อมมีสิทธิต่าง ๆ ที่พึงได้รับ เช่นสิทธิในการได้รับการรักษาด้วยความเคารพและการไม่ละเมิดความเป็นส่วนตัวในฐานะผู้ป่วย เจ้าหน้าที่ศูนย์ฟอกเลือดเคยแสดงหนังสือยินยอมที่แจ้งข้อมูลเกี่ยวกับสิทธิที่ท่านพึงได้รับ พร้อมทั้งให้ลงนามรับทราบในหนังสือยินยอมดังกล่าวหรือไม่

🞎^1^ ใช่

🞎^2^ ไม่ใช่

29. เจ้าหน้าที่ศูนย์ฟอกเลือดได้ทวนสิทธิผู้ป่วยให้ท่านทราบหรือไม่

🞎^1^ ใช่

🞎^2^ ไม่ใช่

30. เจ้าหน้าที่ศูนย์ฟอกเลือดเคยแจ้งให้ท่านทราบถึงวิธีปฏิบัติหากเกิดปัญหาด้านสุขภาพเมื่ออยู่ที่บ้านหรือไม่

🞎^1^ ใช่

🞎^2^ ไม่ใช่

31. เจ้าหน้าที่ศูนย์ฟอกเลือดเคยแจ้งให้ทราบถึงวิธีออกจากเครื่องฟอกเลือดเมื่อเกิดเหตุฉุกเฉินที่ศูนย์หรือไม่

🞎^1^ ใช่

🞎^2^ ไม่ใช่

32. ท่านจะให้คะแนนเจ้าหน้าที่ศูนย์ฟอกเลือดในระดับใด

(โดย 0 เท่ากับระดับแย่ที่สุด และ 10 เท่ากับระดับดีที่สุด)

🞎 0 แย่ที่สุด

🞎 1

🞎 2

🞎 3

🞎 4

🞎 5

🞎 6

🞎 7

🞎 8

🞎 9

🞎 10 ดีที่สุด

หมวดที่ 3 ศูนย์ฟอกเลือด

33. ในช่วง 3 เดือนที่ผ่านมา ท่านใดรับการฟอกเลือดภายใน 15 นาที เมื่อท่านมาถึงศูนย์ฟอกเลือดตรงเวลานัดหมาย บ่อยเพียงใด

🞎^1^ ไม่เคย

🞎^2^ บางครั้ง

🞎^3^ บ่อย

🞎^4^ ประจำ

34. ในช่วง 3 เดือนที่ผ่านมา ท่านรู้สึกว่าศูนย์ฟอกเลือดได้รักษาความสะอาดโดยรวม บ่อยเพียงใด

🞎^1^ ไม่เคย

🞎^2^ บางครั้ง

🞎^3^ บ่อย

🞎^4^ ประจำ

35. ท่านจะให้คะแนนศูนย์ฟอกเลือดในระดับใด

(โดย 0 เท่ากับระดับแย่ที่สุด และ 10 เท่ากับระดับดีที่สุด)

🞎 0 แย่ที่สุด

🞎 1

🞎 2

🞎 3

🞎 4

🞎 5

🞎 6

🞎 7

🞎 8

🞎 9

🞎 10 ดีที่สุด

หมวดที่ 4 การรักษาพยาบาล

คำถามต่อไปนี้เกี่ยวกับรักษาพยาบาลที่ท่านได้รับท่านในช่วง 12 เดือนที่ผ่านมา

36. ผู้ป่วยโรคไตสามารถรับการรักษาโดยการปลูกถ่ายไต รับฟอกเลือดที่ศูนย์ หรือ ล้างไตทางช่องท้อง ในช่วง 12 เดือนที่ผ่านมา แพทย์โรคไตของท่านหรือเจ้าหน้าที่ศูนย์ฟอกเลือดได้เคยให้ข้อมูลเกี่ยวกับการรักษาที่เหมาะสมกับท่านเท่าที่ท่านต้องการหรือไม่

🞎^1^ ใช่

🞎^2^ ไม่ใช่

37. ท่านเข้าเกณฑ์การปลูกถ่ายไตหรือไม่

🞎^1^ ใช่ 🡪 **หากตอบข้อนี้ ให้ข้ามไปข้อ 39**

🞎^2^ ไม่ใช่

🞎^3^ ไม่ทราบ 🡪 **หากตอบข้อนี้ ให้ข้ามไปข้อ 39**

38. ในช่วง 12 เดือนที่ผ่านมานี้ แพทย์โรคไตหรือเจ้าหน้าที่ศูนย์ฟอกเลือด ได้เคยอธิบายถึงสาเหตุที่ท่านไม่สามารถรับการปลูกถ่ายไตหรือไม่

🞎^1^ ใช่

🞎^2^ ไม่ใช่

39. ในช่วง 12 เดือนที่ผ่านมา แพทย์โรคไตหรือเจ้าหน้าที่ศูนย์ฟอกเลือดได้เคยพูดคุยกับท่านเกี่ยวกับการล้างไตทางช่องท้อง หรือไม่

🞎^1^ ใช่

🞎^2^ ไม่ใช่

40. ในช่วง 12 เดือนที่ผ่านมา ท่านได้มีส่วนร่วมในการเลือกการรักษาโรคไตที่เหมาะสมสำหรับตัวเองหรือไม่

🞎^1^ ใช่

🞎^2^ ไม่ใช่

41. ในช่วง 12 เดือนที่ผ่านมา ท่านเคยรู้สึกไม่พอใจกับการรักษาจากศูนย์ฟอกเลือดหรือแพทย์โรคไตของท่านหรือไม่

🞎^1^ ใช่

🞎^2^ ไม่ใช่ 🡪 **หากตอบข้อนี้ ให้ข้ามไปข้อ 45**

42. **จากข้อ 41** ในช่วง 12 เดือนที่ผ่านมา ท่านได้เคยพูดคุยกับบุคคลอื่นถึงความไม่พอใจที่มีต่อเจ้าหน้าที่ศูนย์ฟอกเลือดหรือไม่

🞎^1^ ใช่

🞎^2^ ไม่ใช่ 🡪 **หากตอบข้อนี้ ให้ข้ามไปข้อ 45**

43. ในช่วง 12 เดือนที่ผ่านมาท่านรู้สึกพอใจกับวิธีการจัดการปัญหาด้านการรักษาของเจ้าหน้าที่ศูนย์ฟอกเลือดบ่อยเพียงใด

🞎^1^ ไม่เคย

🞎^2^ บางครั้ง

🞎^3^ บ่อย

🞎^4^ ประจำ

44. สำนักงานสาธารณสุขประจำจังหวัดที่ท่านอาศัย สามารถตรวจสอบคุณภาพการให้บริการของศูนย์ฟอกเลือดได้ ในช่วง 12 เดือนที่ผ่านมา ท่านเคยได้ร้องเรียนไปยังหน่วยงานดังกล่าวหรือไม่

🞎^1^ ใช่

🞎^2^ ไม่ใช่ 🡪 **หากตอบข้อนี้ ให้ข้ามไปข้อ 45**

หมวดที่ 5 เกี่ยวกับตัวท่าน

45. ท่านให้คะแนนสุขภาพของตัวเองโดยรวมในระดับใด

🞎^1^ เยี่ยมยอด

🞎^2^ ดีมาก

🞎^3^ ดี

🞎^4^ พอใช้

🞎^5^ แย่

46. ท่านให้คะแนนสุขภาพจิต และอารมณ์ โดยรวมของท่านในระดับใด

🞎^1^ เยี่ยมยอด

🞎^2^ ดีมาก

🞎^3^ ดี

🞎^4^ พอใช้

🞎^5^ แย่

47. ท่านกำลังรับการรักษาโรคความดันโลหิตสูงหรือไม่?

🞎^1^ ใช่

🞎^2^ ไม่ใช่

48. ท่านกำลังรับการรักษาโรคเบาหวานหรือภาวะน้ำตาลในเลือดสูงหรือไม่

🞎^1^ ใช่

🞎^2^ ไม่ใช่

49. ท่านกำลังรับการรักษาโรคหัวใจหรือโรคเกี่ยวกับหัวใจหรือไม่

🞎^1^ ใช่

🞎^2^ ไม่ใช่

50. กรุณาเลือกช่วงอายุของท่าน

🞎^1^ 18 - 24 ปี

🞎^2^ 25 - 34 ปี

🞎^3^ 35 - 44 ปี

🞎^4^ 45 - 54 ปี

🞎^5^ 55 - 64 ปี

🞎^6^ 65 - 74 ปี

🞎^7^ 75 ปี ขึ้นไป

51. กรุณาเลือกเพศของท่าน

🞎^1^ ชาย

🞎^2^ หญิง

52. กรุณาเลือกระดับการศึกษาของท่าน

🞎^1^ ประถมศึกษา

🞎^2^ มัธยมต้น

🞎^3^ มัธยมปลาย

🞎^4^ ปริญญาตรี

🞎^5^ สูงกว่าปริญญาตรี

53. ท่านเป็นบุคคลต่างด้าวหรือไม่

🞎^1^ ใช่

🞎^2^ ไม่ใช่

54. กรุณาเลือกเชื้อชาติของท่าน

🞎^1^ ไทย

🞎^2^ อื่นๆ

55. ท่านใช้ภาษาใดเป็นภาษาหลักในการสื่อสารกับบุคคลอื่นที่บ้าน

🞎^1^ ภาษาไทย

🞎^2^ ภาษาไทย และภาษาอื่นๆ อย่างเท่า ๆ กัน

🞎^3^ อื่น ๆ โปรดระบุ________________________

56. มีผู้อื่นช่วยท่านทำแบบสำรวจนี้หรือไม่

🞎^1^ ใช่

🞎^2^ ไม่ใช่ 🡪 **หากตอบข้อนี้ ให้หยุดตรงนี้**

57. บุคคลใดช่วยท่านทำแบบสำรวจนี้

🞎^1^ ครอบครัว

🞎^2^ เพื่อน

🞎^3^ เจ้าหน้าที่ศูนย์ฟอกไต

🞎^4^ อื่นๆ โปรดระบุ________________________

58. บุคคลจากข้อ 57 ได้ช่วยเหลือท่านอย่างไร เลือกได้มากกว่า 1 ข้อ

🞎^1^ อ่านคำถามให้ฟัง

🞎^2^ เขียนคำตอบตามคำบอก

🞎^3^ ตอบคำถามแทน

🞎^4^ แปลคำถามเป็นภาษาของท่าน

🞎^5^ อื่นๆ โปรดระบุ________________________

ขอบพระคุณที่ให้ความร่วมมือ

**Figure S1** Trends in the incidence rate of ESKD in Thailand, 2003-2016 (Adapted from United States Renal Data System: 2018 USRDS annual data report. Volume II: International comparisons. Available at: <https://www.niddk.nih.gov/-/media/Files/USRDS/2018-Previous-ADR/Reference-Tables/esrd_ref_n_international_2018.xlsx>. Accessed November 22, 2025.)

**Figure S2** Flow diagram of the study participants

*Patient who needed as assistance is defined as a patient with Karnofsky performance scale <50

**Table S1** Patient characteristics

| Characteristics | N (%) (Total=189) |
| --- | --- |
| **Age (years old)**  18 – 34  35 – 54  55 – 74  75 or more | 12 (6.35)  53 (28.04)  105 (55.56)  19 (10.05) |
| **Sex**  Male  Female | 107 (56.61)  82 (43.39) |
| BMI (kg/m^2^)  <18.5  18.5 – 22.9  23 – 25  >25 | 23 (12.17)  72 (38.10)  37 (19.58)  57 (30.16) |
| Dialysis Vintage  3 months – less than 1 year  1 – less than 5 years  5 years or more | 31 (16.40)  100 (52.91)  58 (30.69) |
| Education  Elementary School  Junior high school  Senior high school  Bachelor  Master’s degree or more | 56/187 (29.95)  17/187 (9.09)  30/187 (16.04)  63/187 (33.69)  21/187 (11.23) |
| Survey response  Self-complete  Reading the question  Writing down the given answer  Translating the question into local language | 143 (75.66)  43 (22.75)  25 (13.23)  4 (2.12) |
| Urine output  ≥200 ml/d  <200 ml/d | 87 (46.03)  102 (53.97) |
| Kt/V  ≤1.2  >1.2 | 21/188 (11.17)  167/188 (88.83) |
| Hemoglobin level  <10 mg/dL  10–11.9 mg/dL  ≥12 mg/dL | 80 (42.33)  92 (48.68)  17 (8.99) |
| Albumin level  <2.5 mg/dL  2.5-<3.5 mg/dL  ≥3.5 mg/dL | 0  18 (9.52)  171 (90.48) |
| PTH level  <30  30 – 500  >500 | 12 (6.35)  128 (67.72)  49 (25.93) |
| 9-THAI Survey  Physical, mean±SD [n]  Mental, mean±SD [n] | 33.38±19.41 [188]  35.37±16.68 [181] |

**Table S2** Ceiling and floor of the Thai CAHPS-ICH

| **Thai CAHPS-ICH** | **Baseline** | | **1 month** | |
| --- | --- | --- | --- | --- |
|  | **%Ceiling effect** | **%Floor effect** | **%Ceiling effect** | **%Floor effect** |
| Q3 Doctor listens carefully | 59.57 | 1.06 | 52.69 | 0.60 |
| Q4 Doctor explains things | 38.67 | 2.76 | 42.17 | 1.81 |
| Q5 Doctor shows respect | 47.49 | 0 | 44.85 | 0.61 |
| Q6 Doctor spends enough time | 42.54 | 0.55 | 48.19 | 0 |
| Q7 Doctor cared about you | 48.62 | 1.10 | 46.99 | 0.60 |
| Q8 Nephrologists’ communication and caring | 39.89 | 0 | 43.71 | 0 |
| Q10 Staff listen carefully | 64.84 | 0 | 59.52 | 0 |
| Q11 Staff explain in a way that is easy to understand | 55.25 | 0 | 54.76 | 0 |
| Q12 Staff show respect | 51.65 | 0 | 55.36 | 0 |
| Q13 Staff spend enough time | 57.07 | 0 | 52.69 | 0.60 |
| Q14 Staff cared about you | 57.84 | 0 | 55.95 | 0 |
| Q15 Staff makes you comfortable | 61.41 | 0.54 | 62.50 | 2.38 |
| Q21 Staff insert needle without pain | 34.07 | 1.65 | 36.42 | 0 |
| Q22 Staff check you closely | 57.61 | 0.54 | 52.07 | 1.18 |
| Q24 Staff manage problems | 12.07 | 0 | 17.18 | 0.61 |
| Q25 Staff professional | 64.77 | 0 | 56.36 | 1.82 |
| Q27 Staff explain tests | 32.42 | 4.95 | 31.52 | 4.24 |
| Q32 Quality of Dialysis Center Care and Operations | 46.70 | 0 | 46.43 | 0 |
| Q33 On machine within 15 min | 32.78 | 13.89 | 35.33 | 10.78 |
| Q34 Center clean | 67.21 | 0.55 | 65.87 | 0 |
| Q35 Providing Information to Patients | 48.91 | 0 | 50.90 | 0 |
| Q43 Satisfied with way problems handled | 1.14 | 0 | 1.23 | 1.23 |

**Table S3** Correlations between Scales and Global Rating Scales

| Scale domain | **Doctor**  **rating scale** | **Staff**  **rating scale** | **Center**  **rating scale** |
| --- | --- | --- | --- |
| Nephrologists’ Communication and Caring (NCC) | 0.60*** | 0.45*** | 0.38*** |
| Quality of Dialysis Center Care and Operations (QoC) | 0.37*** | 0.65*** | 0.58*** |
| Providing Information to Patients (PI) | 0.17* | 0.26** | 0.31*** |
| *p < 0.05, **p < 0.01, ***p < 0.001 | | | |

**Table S4** Confirmatory Factor Analysis of the hypothesized three-composite structure

| **Item** | **Standardized Factor Loadings** | **95% Confidence Interval** | **P value** |
| --- | --- | --- | --- |
| **Nephrologists’ communication and caring (n=166)^a^**  Q3 Doctor listens carefully  Q4 Doctor explains things  Q5 Doctor shows respect  Q6 Doctor spends enough time  Q7 Doctor cared about you  Q9 Doctor seemed informed | 0.7860  0.8554  0.7884  0.7851  0.8083  -0.0720 | 0.7167, 0.8553  0.8008, 0.9099  0.7201, 0.8567  0.7142, 0.8559  0.7420, 0.8747  -0.2314, 0.0874 | <0.0001  <0.0001  <0.0001  <0.0001  <0.0001  0.376 |
| **Quality of Dialysis Center Care and Operations (n = 142)^b^**  Q10 Staff listen carefully  Q11 Staff explain in a way that is easy to understand  Q12 Staff show respect  Q13 Staff spend enough time  Q14 Staff cared about you  Q15 Staff makes you comfortable  Q16 Staff keep information private  Q17 Comfortable asking staff  Q21 Staff insert needle without pain  Q22 Staff check you closely  Q24 Staff manage problems  Q25 Staff professional  Q26 Staff discuss diet  Q27 Staff explain tests  Q33 On machine within 15 min  Q34 Center clean  Q43 Satisfied with way problems handled | 0.7491  0.7851  0.8604  0.8121  0.8716  0.8186  -0.2649  -0.1435  0.1040  0.6953  0.0116  0.6349  -0.2749  0.5449  0.3104  0.5749  -0.2789 | 0.6696, 0.8285  0.7146, 0.8557  0.8106, 0.9101  0.7488, 0.8753  0.8240, 0.9192  0.7577, 0.8795  -0.4234, -0.1065  -0.3099, 0.0229  -0.0640, 0.2720  0.6043, 0.7862  -0.1581, 0.1813  0.5308, 0.7389  -0.4327. -0.1170  0.4237, 0.6662  0.1565, 0.4643  0.4591, 0.6906  -0.4359, -0.1219 | <0.0001  <0.0001  <0.0001  <0.0001  <0.0001  <0.0001  0.001  0.091  0.225  <0.0001  0.893  <0.0001  0.001  <0.0001  <0.0001  <0.0001  <0.0001 |
| **Providing Information to Patients (n = 150)^c^**  Q19 Know how to care of access site  Q28 Staff give information on patient rights  Q29 Staff review patient rights  Q30 Staff told you what to do if health problem at home  Q31 Staff told you how to get off machine if emergency  Q36 Doctor/staff talk about which treatment is right for you  Q38 Doctor/staff explain why not eligible for transplant  Q39 Doctor/staff talk about peritoneal dialysis  Q40 Involved in choosing treatment | 0.3290  0.5949  0.6582  0.2081  0.5996  0.1772  0.1720  0.3635  0.2546 | 0.1495, 0.5085  0.4384, 0.7515  0.5063, 0.8101  0.0214, 0.3948  0.4430, 0.7563  -0.0142, 0.3685  -0.0187, 0.3628  0.1855, 0.5414  0.0624, 0.4469 | <0.0001  <0.0001  <0.0001  0.029  <0.0001  0.070  0.077  <0.0001  0.009 |
| ^a^Likelihood ratio test of model versus saturated: Root mean squared error of approximation (RMSEA), 90% CI = 0.137 (0.093, 0.185); comparative fit index (CFI) = 0.945; standardized root mean squared residual (SRMR) = 0.041  ^b^Likelihood ratio test of model versus saturated: RMSEA, 90% CI = 0.098 (0.083, 0.113); CFI = 0.844; SRMR = 0.071  ^c^Likelihood ratio test of model versus saturated: RMSEA, 90% CI = 0.066 (0.027, 0.099); CFI = 0.843; SRMR = 0.070 | | | |

**Table S5** Comparison of CFA model Fit Indices (NCC Domain)

| Model | **RMSEA** | **CFI** | **SRMR** |
| --- | --- | --- | --- |
| Original Model (with Q9) | 0.137 | 0.945 | 0.041 |
| Sensitivity Model (without Q9) | 0.305 | 0.933 | 0.046 |
| RMSEA = Root mean squared error of approximation; CFI = comparative fit index; SRMR = standardized root mean squared residual | | | |

**Table S6** Item-scale correlations of the Thai CAHPS-ICH composites

| **Items** | **Scales** | | |
| --- | --- | --- | --- |
|  | **Doctor rating** | **Staff rating** | **Center rating** |
| Q3 Doctor listens carefully (n = 176)  Q4 Doctor explains things (n = 175)  Q5 Doctor shows respect (n = 173)  Q6 Doctor spends enough time (n = 175)  Q7 Doctor cared about you (n = 175)  Q9 Doctor seemed informed (n = 163) | 0.51^a^  0.58^a^  0.55^a^  0.54^a^  0.55^a^  -0.03 | 0.42^a^  0.42^a^  0.44^a^  0.37^a^  0.48^a^  -0.07 | 0.38^a^  0.43^a^  0.41^a^  0.42^a^  0.55^a^  <-0.001 |
| Q10 Staff listen carefully (n = 174)  Q11 Staff explain in a way that is easy to understand (n = 174)  Q12 Staff show respect (n = 172)  Q13 Staff spend enough time (n = 173)  Q14 Staff cared about you (n = 174)  Q15 Staff makes you comfortable (n = 173)  Q16 Staff keep information private (n = 168)  Q17 Comfortable asking staff (n = 173)  Q21 Staff insert needle without pain (n = 171)  Q22 Staff check you closely (n = 173)  Q24 Staff manage problems (n = 168)  Q25 Staff professional (n = 170)  Q26 Staff discuss diet (n = 174)  Q27 Staff explain tests (n = 176)  Q33 On machine within 15 min (n = 174)  Q34 Center clean (n = 175)  Q43 Satisfied with way problems handled (n = 167) | 0.28^a^  0.33^a^  0.39^a^  0.38^a^  0.34^a^  0.37^a^  0.02  -0.08  0.07  0.33^a^  -0.04  0.33^a^  -0.24^a^  0.34^a^  0.22^a^  0.33^a^  -0.15^a^ | 0.48^a^  0.50^a^  0.59^a^  0.51^a^  0.57^a^  0.54^a^  -0.08  -0.03 0.16^a^  0.54^a^  -0.04  0.47^a^  -0.25^a^  0.49^a^  0.25^a^  0.46^a^  -0.27^a^ | 0.37^a^  0.44^a^  0.48^a^  0.39^a^  0.42^a^  0.46^a^  -0.04  0.02  0.15  0.47^a^  -0.06  0.34^a^  -0.14  0.37^a^  0.25^a^  0.55^a^  -0.22^a^ |
| Q19 Know how to care of access site (n = 173)  Q28 Staff give information on patient rights (n = 169)  Q29 Staff review patient rights (n = 167)  Q30 Staff told you what to do if health problem at home  (n = 176)  Q31 Staff told you how to get off machine if emergency (n = 173)  Q36 Doctor/staff talk about which treatment is right for you  (n = 165)  Q38 Doctor/staff explain why not eligible for transplant (n = 171)  Q39 Doctor/staff talk about peritoneal dialysis (n = 168)  Q40 Involved in choosing treatment (n = 168) | -0.06  -0.25^a^  -0.30^a^  -0.13  -0.03  -0.10  -0.14  -0.20^a^  -0.08 | -0.20^a^  -0.30^a^  -0.31^a^  -0.24^a^  -0.11  -0.04  -0.14  -0.20^a^  -0.07 | -0.21^a^  -0.22^a^  -0.32^a^  -0.15^a^  -0.15^a^  -0.06  -0.13  -0.21^a^  -0.01 |
| ^a^p < 0.05 | | | |

**Table S7** Internal Consistency Reliability and Item-level Reliability Analysis

| **Nephrologists’ communication and caring** |  | **Alpha=0.8614** |
| --- | --- | --- |
|  | **Corrected-item total correlation** | **Cronbach’s alpha if item deleted** |
| Q3 Doctor listens carefully  Q4 Doctor explains things  Q5 Doctor shows respect  Q6 Doctor spends enough time  Q7 Doctor cared about you  Q9 Doctor seemed informed | 0.5697  0.7234  0.7030  0.6431  0.7100  0.0686 | 0.8259  0.8106  0.8185  0.8275  0.8175  0.8984 |
| **Quality of Dialysis Center Care and Operations** | | **Alpha=0.7816** |
| Q10 Staff listen carefully  Q11 Staff explain in a way that is easy to understand  Q12 Staff show respect  Q13 Staff spend enough time  Q14 Staff cared about you  Q15 Staff makes you comfortable  Q16 Staff keep information private  Q17 Comfortable asking staff  Q21 Staff insert needle without pain  Q22 Staff check you closely  Q24 Staff manage problems  Q25 Staff professional  Q26 Staff discuss diet  Q27 Staff explain tests  Q33 On machine within 15 min  Q34 Center clean  Q43 Satisfied with way problems handled | 0.6430  0.6186  0.6788  0.6622  0.7210  0.6971  0.2004  0.1607  0.1616  0.5928  -0.0085  0.5490  0.2753  0.4892  0.3043  0.5280  0.1755 | 0.7503  0.7533  0.7490  0.7518  0.7491  0.7495  0.7814  0.7829  0.8138  0.7550 0.8305  0.7617  0.7795  0.7594  0.7800  0.7647  0.7832 |
| **Providing Information to Patients** | | **Alpha=0.5303** |
| Q19 Know how to care of access site  Q28 Staff give information on patient rights  Q29 Staff review patient rights  Q30 Staff told you what to do if health problem at home  Q31 Staff told you how to get off machine if emergency  Q36 Doctor/staff talk about which treatment is right for you  Q38 Doctor/staff explain why not eligible for transplant  Q39 Doctor/staff talk about peritoneal dialysis  Q40 Involved in choosing treatment | 0.1267  0.2663  0.2903  0.1712  0.4178  0.2561  0.1458  0.3189  0.2888 | 0.5253  0.4954  0.4826  0.5221  0.4404  0.4992  0.5920  0.4777  0.4791 |

**Table S8** Intraclass correlation

| **Items** | **ICC** | **95%CI** | **P value** |
| --- | --- | --- | --- |
| Q3 Doctor listens carefully | 0.66 | 0.54 - 0.75 | <0.001 |
| Q4 Doctor explains things | 0.68 | 0.67 - 0.77 | <0.001 |
| Q5 Doctor shows respect | 0.66 | 0.53 - 0.75 | <0.001 |
| Q6 Doctor spends enough time | 0.59 | 0.43 - 0.70 | <0.001 |
| Q7 Doctor cared about you | 0.56 | 0.40 - 0.68 | <0.001 |
| Q8 Nephrologists’ communication and caring^2^ | 0.64 | 0.51 - 0.73 | <0.001 |
| Q9 Doctor seemed informed^1^ | 0.48 | 0.28 - 0.62 | <0.001 |
| Q10 Staff listen carefully | 0.70 | 0.59 - 0.78 | <0.001 |
| Q11 Staff explain in a way that is easy to understand | 0.65 | 0.52 - 0.74 | <0.001 |
| Q12 Staff show respect | 0.70 | 0.60 - 0.78 | <0.001 |
| Q13 Staff spend enough time | 0.66 | 0.53 - 0.75 | <0.001 |
| Q14 Staff cared about you | 0.62 | 0.49 - 0.72 | <0.001 |
| Q15 Staff makes you comfortable | 0.63 | 0.49 - 0.72 | <0.001 |
| Q16 Staff keep information private^1^ | 0.22 | -0.07 - 0.43 | 0.061 |
| Q17 Comfortable asking staff^1^ | -0.06 | -0.44 - 0.22 | 0.638 |
| Q21 Staff insert needle without pain | 0.85 | 0.79 - 0.89 | <0.001 |
| Q22 Staff check you closely | 0.57 | 0.42 - 0.68 | <0.001 |
| Q24 Staff manage problems | 0.50 | 0.31 - 0.63 | <0.001 |
| Q25 Staff professional | 0.61 | 0.47 - 0.72 | <0.001 |
| Q26 Staff discuss diet^1^ | 0.30 | 0.05 - 0.49 | 0.012 |
| Q27 Staff explain tests | 0.61 | 0.47 - 0.71 | <0.001 |
| Q32 Quality of Dialysis Center Care and Operations^2^ | 0.70 | 0.60 - 0.78 | <0.001 |
| Q33 On machine within 15 min | 0.65 | 0.52 - 0.74 | <0.001 |
| Q34 Center clean | 0.54 | 0.37 - 0.66 | <0.001 |
| Q35 Providing Information to Patients^2^ | 0.79 | 0.72 - 0.85 | <0.001 |
| Q43 Satisfied with way problems handled | 0.19 | -0.12 - 0.41 | 0.105 |
| Q19 Know how to care of access site^1^ | 0.28 | 0.02 - 0.47 | 0.020 |
| Q28 Staff give information on patient rights^1^ | 0.13 | -0.19 - 0.37 | 0.190 |
| Q29 Staff review patient rights^1^ | 0.68 | 0.55 - 0.77 | <0.001 |
| Q30 Staff told you what to do if health problem at home^1^ | 0.58 | 0.42 - 0.69 | <0.001 |
| Q31 Staff told you how to get off machine if emergency^1^ | 0.57 | 0.41 - 0.69 | <0.001 |
| Q36 Doctor/staff talk about which treatment is right for you^1^ | 0.34 | 0.09 - 0.52 | 0.006 |
| Q38 Doctor/staff explain why not eligible for transplant^1^ | 0.70 | 0.59 - 0.79 | <0.001 |
| Q39 Doctor/staff talk about peritoneal dialysis^1^ | 0.47 | 0.27 - 0.61 | <0.001 |
| Q40 Involved in choosing treatment^1^ | 0.44 | 0.23 - 0.59 | <0.001 |
| ^1^Yes/No question  ^2^Global rating scale | | | |
